# The TNFα/TNFR2 axis mediates natural killer cell proliferation by promoting aerobic glycolysis

**DOI:** 10.1038/s41423-023-01071-4

**Published:** 2023-08-09

**Authors:** Abrar Ul Haq Khan, Alaa Kassim Ali, Bryan Marr, Donghyeon Jo, Simin Ahmadvand, Claire Fong-McMaster, Saeedah Musaed Almutairi, Lisheng Wang, Subash Sad, Mary-Ellen Harper, Seung-Hwan Lee

**Affiliations:** 1 Department of Biochemistry, Microbiology, and Immunology, Faculty of Medicine, University of Ottawa, Ottawa, ON Canada; 2 The University of Ottawa Centre for Infection, Immunity, and Inflammation, Ottawa, ON Canada; 3 Botany and Microbiology Department, College of Sciences, King Saud University, Riyadh, Saudi Arabia

**Keywords:** Natural killer cells, TNFα, TNFR2, Glycolysis, Proliferation, Murine cytomegalovirus, Innate immunity, Tumour-necrosis factors, Cell growth, Infection

## Abstract

Natural killer (NK) cells are predominant innate lymphocytes that initiate the early immune response during infection. NK cells undergo a metabolic switch to fuel augmented proliferation and activation following infection. Tumor necrosis factor-alpha (TNFα) is a well-known inflammatory cytokine that enhances NK cell function; however, the mechanism underlying NK cell proliferation in response to TNFα is not well established. Here, we demonstrated that upon infection/inflammation, NK cells upregulate the expression of TNF receptor 2 (TNFR2), which is associated with increased proliferation, metabolic activity, and effector function. Notably, IL-18 can induce TNFR2 expression in NK cells, augmenting their sensitivity toward TNFα. Mechanistically, TNFα-TNFR2 signaling upregulates the expression of CD25 (IL-2Rα) and nutrient transporters in NK cells, leading to a metabolic switch toward aerobic glycolysis. Transcriptomic analysis revealed significantly reduced expression levels of genes involved in cellular metabolism and proliferation in NK cells from TNFR2 KO mice. Accordingly, our data affirmed that genetic ablation of TNFR2 curtails CD25 upregulation and TNFα-induced glycolysis, leading to impaired NK cell proliferation and antiviral function during MCMV infection in vivo. Collectively, our results delineate the crucial role of the TNFα-TNFR2 axis in NK cell proliferation, glycolysis, and effector function.

## Introduction

Tumor necrosis factor-alpha (TNFα) is considered one of the pleiotropic cytokines described in mammals [1]. It plays a critical role in the activation, death, and proliferation of both immune and non-immune cells. TNFα is initially expressed as a transmembrane protein assembled into a homotrimer structure. Membrane-bound TNFα is cleaved by disintegrin and metalloprotease 17 (ADAM17) and is released as a soluble form of the TNFα homotrimer [2, 3]. Notably, both membrane-bound and soluble TNFα can induce the pleiotropic function of TNFα in auto/para/juxta/endocrine modes of action.

TNFα biology is further complicated by the two TNFα receptors. Both membrane-bound and soluble TNFα can bind to the structurally related but functionally distinct receptors, TNFR1 (p55/60) and TNFR2 (p75/80), with various affinities [3, 4]. TNFR1 is ubiquitously expressed in immune and non-immune cells such as stromal cells, fibroblasts, and endothelial cells [5], whereas TNFR2 is expressed in several cell types, including Treg cells, activated T lymphocytes, endothelial cells, and neural cells [3, 6]. As both receptors lack intrinsic enzyme activity, intracellular signal transduction depends on cytosolic molecules recruited upon ligand binding. Briefly, TNFR1 contains a death domain that interacts with TNFR1-associated death-domain protein, inducing signals for the activation of the nuclear factor-kB (NF-κB) pathway or cell death pathway depending on caspase activation [2]. In contrast, TNFR2 interacts with TNFR-associated factors without a death domain and favors cell proliferation and activation by triggering the NF-κB pathway [3, 7]. Signals from TNFR1 and TNFR2 are interconnected and context dependent, inducing various cell responses to TNFα, including cell activation, proliferation, and cell survival/death [1–3]. Thus, characterizing the effect of TNFα in the context of either TNFR1 or TNFR2 in a particular immune cell is critical to dissect the role of TNFα and to exploit the TNFα signal in therapeutics.

Natural killer (NK) cells are innate lymphocytes that play an essential role in the early response to microbial infections. Following infection/inflammation, NK cells are the first to proliferate to serve as the principal cytotoxic cells [8, 9]. NK cells rapidly undergo metabolic reprogramming during infection and inflammation to support their expansion and differentiation into potent effector NK cells [10]. We and others have reported that IL-2/15 activates mTORC1, which is a critical metabolic sensor required for glycolytic reprogramming through the upregulation of several glycolytic enzymes and GLUT1 [11–13]. In immune cells, including NK cells, the mTORC1 pathway is hardwired to acquire effector functions, which is exemplified by impaired effector functions in mTORC1-deficient or rapamycin-treated mice [12, 13]. Metabolic changes are usually accompanied by increased expression levels of nutrient transporters, leading to increased nutrient uptake. We have also identified the immunometabolic role of IL-18 in upregulating nutrient transporters in NK cells, thereby supporting metabolic changes [14]. During murine cytomegalovirus (MCMV) infection, it is well characterized that several cytokines, such as IL-12, IL-18, and IL-15, induce NK cell proliferation [15–18]. In addition, the activating NK cell receptor Ly49H recognizes the MCMV-m157 glycoprotein expressed on infected cells, inducing the killing of infected cells and preferential proliferation of the Ly49H^+^ subset [19].

Previous studies have shown that TNFα signaling through TNFR2 enhances NK cell function [20, 21]. However, the mechanism by which TNFR2 activates NK cells is not fully understood. To the best of our knowledge, the present study is the first to identify the crucial role of TNFα-TNFR2 axis that can enhance NK cell proliferation by switching their metabolic activity to aerobic glycolysis.

## Results

### Following MCMV- or LPS-induced inflammation, NK cell activation is accompanied by the upregulation of TNFR2

**Fig. 1:**
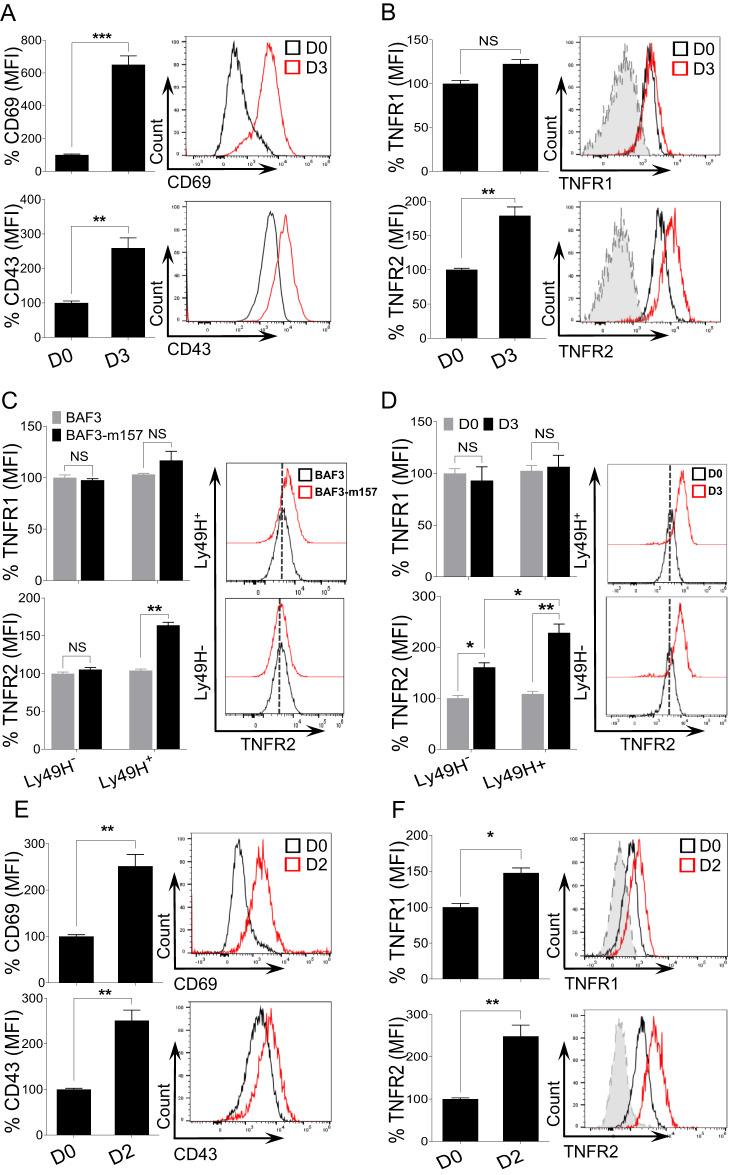
TNFR2 is highly expressed on NK cells upon MCMV- or LPS-induced inflammation. C57BL/6 mice were either left untreated or intraperitoneally infected with 3000 PFU MCMV and analyzed on the indicated days. Representative graphs describe the mean fluorescence intensity (MFI) of (**A**) CD69 and CD43 and (**B**) TNFR1 and TNFR2 expression on NK cells from the spleens of naive (D0) or MCMV-infected mice on Day 3 (D3) post-infection. **C** NK cells were enriched from the spleens of naive C57BL/6 mice and co-cultured with either BAF3 or BAF3-m157 cells for 18 h, and the expression of TNFR1 and TNFR2 was measured on NK cells. **D** C57BL/6 mice were either left untreated or infected with MCMV and analyzed on the indicated day. Representative graphs depict the MFI of TNFR1 and TNFR2 expression on NK cells from the spleen of naive (D0) or MCMV-infected mice on Day 3 (D3). C57BL/6 mice were either left untreated or intraperitoneally injected with LPS and analyzed on the indicated days. Representative graphs depict the MFI of (**E**) CD69 and CD43 and (**F**) TNFR1 and TNFR2 expression on NK cells from the spleens of naive (D0) or LPS-treated mice on Day 2 post-injection (D2). The MFI expression is presented as a percentage relative to the MFI of control mice as 100. Data are from one experiment representative of three independent experiments, with at least three mice per group. Data represent the mean ± SD. *p < 0.05; **p < 0.01; ***p < 0.001; ns nonsignificant

To investigate the role of TNFα signaling in NK cell activation, we utilized an in vivo model of acute infection by challenging C57BL/6 (B6) mice with MCMV. Three days post-infection (D3), splenic NK cells were analyzed for their expression of the two TNF receptors, TNFR1 and TNFR2. A gating strategy for NK cell identification is shown in Supplementary Fig. 1. NK cells from MCMV-infected mice were highly activated, as indicated by their upregulation of the activation markers CD69 and CD43 on D3 post-infection (Fig. 1A). In infected mice, the expression of TNFR1 on NK cells did not change, while there was a significant upregulation of TNFR2 expression (Fig. 1B).

The activating receptor Ly49H on murine NK cells binds to the MCMV-encoded glycoprotein m157, and this recognition induces the proliferative expansion of the MCMV-specific Ly49H^+^ subset of NK cells [19, 22]. We assessed whether signaling through Ly49H contributes to the upregulation of TNFRs on NK cells. Freshly isolated NK cells from naive B6 mice were co-cultured with either parental BAF3 cells or BAF3 cells expressing m157 glycoprotein (BAF3-m157) ex vivo. Notably, Ly49H^+^ NK cells displayed upregulated expression of TNFR2 when co-cultured with BAF3-m157 cells, and the upregulated expression was strictly Ly49H-dependent (Fig. 1C). We did not observe a significant upregulation of TNFR1 on Ly49H^+^ NK cells co-cultured with BAF-m157 (Fig. 1C). Next, we examined the expression of TNFRs on NK cells from MCMV-infected B6 mice on D3 post-infection with regard to Ly49H expression. Although higher TNFR2 upregulation was observed on Ly49H^+^ NK cells, TNFR2 was also upregulated on Ly49H^-^ NK cells (Fig. 1D), suggesting an alternative pathway for the upregulation of TNFR2 expression on NK cells during MCMV infection. TNFR1 expression was comparable regardless of Ly49H expression and MCMV infection on NK cells.

To determine whether the expression of TNFRs on NK cells is modulated by non-infectious inflammation in vivo, we challenged B6 mice with LPS, and splenic NK cells were analyzed on D2 post-injection. Consistent with the results obtained from the MCMV infection model, NK cells from LPS-treated mice displayed higher expression levels of activation markers than those from control mice (Fig. 1E). Interestingly, we observed that NK cells from LPS-treated mice also upregulated the expression of TNFR1; however, the magnitude of TNFR2 upregulation on NK cells was significantly higher than that of TNFR1 (Fig. 1F). Altogether, these results demonstrate that following MCMV infection or LPS-induced inflammation, the elevated expression levels of TNFR2, and to a lesser extent those of TNFR1, is a phenotypic signature of activated NK cells.

### IL-18 signaling upregulates the expression of TNFR2 on NK cells

**Fig. 2:**
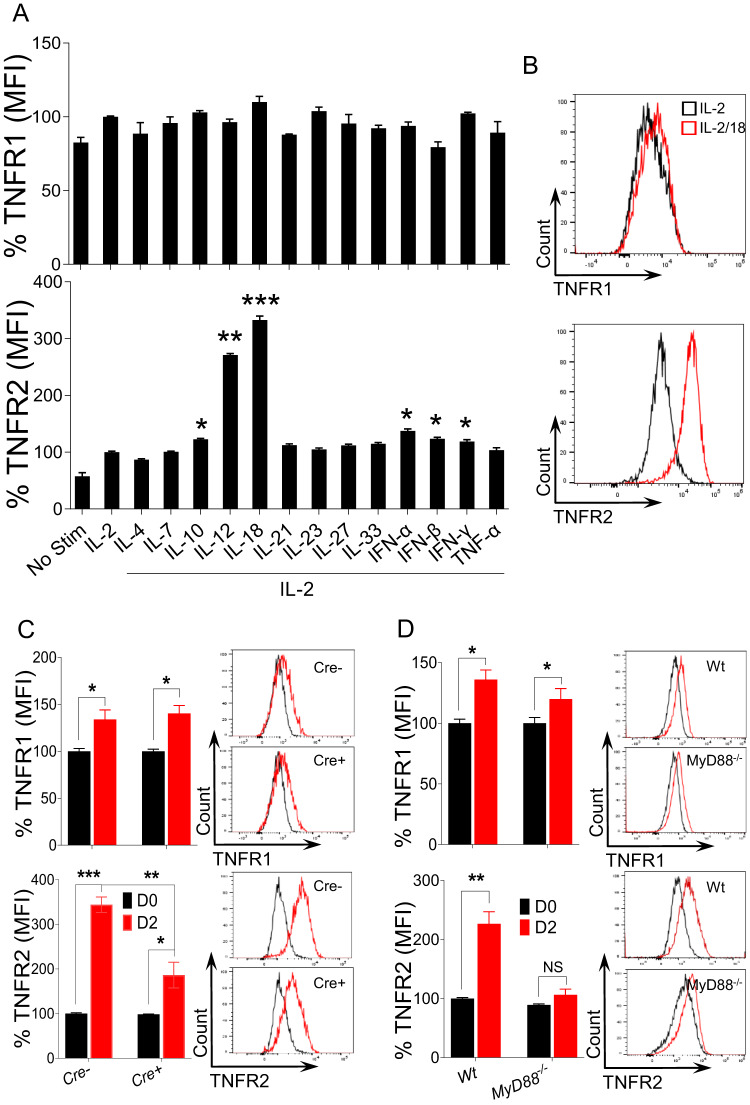
IL-18-MyD88 signaling induces the expression of TNFR2 on NK cells. NK cells were enriched from the spleens of naive C57BL/6 mice and stimulated with the indicated cytokines ex vivo for 24 h. 100 U/ml rhIL-2 was added to maintain NK cell survival. Representative graphs depict the (**A**) MFI and (**B**) protein expression of TNFR1 and TNFR2 on cytokine-stimulated NK cells. Data are from one experiment representative of three independent experiments, with at least two replicates per group. **C** *Il18r1*^*fl/fl*^ (**Cre**−), *NKp46-Cre-Il18r1*^*fl/fl*^ (**Cre**+), and **D** C57BL/6 and *MyD88*^*−/−*^ mice were either left untreated or intraperitoneally injected with LPS and analyzed on the indicated day for the expression of TNFR1 and TNFR2 on splenic NK cells. The MFI expression is presented as a percentage relative to the MFI of control cells as 100. Data are from one experiment representative of two independent experiments, with at least four mice per group. Data represent the mean ± SD. *p < 0.05; **p < 0.01; ***p < 0.001; ns nonsignificant

The upregulation of TNFR2 on NK cells, irrespective of Ly49H expression during MCMV infection, suggested an alternative Ly49H-independent pathway for upregulating TNFR2 on NK cells (Fig. 1D). During MCMV infection, a variety of cytokines are produced, playing immunomodulatory roles in NK cell activity [15, 23]. To determine which cytokines can directly upregulate the expression of TNFR2, we stimulated enriched NK cells with various recombinant cytokines ex vivo. Although different cytokines slightly upregulated the expression of TNFR2, the highest induction was observed following IL-12 and IL-18 stimulation (Fig. 2A). The upregulation of TNFR2 expression on NK cells by IL-12 has been established [21], while IL-18-induced upregulation has not been previously identified, prompting our investigation into this pathway. We previously demonstrated that NK cells have the highest expression of IL-18Rα, indicating that the IL-18-MyD88 pathway plays a critical role in the proliferation and metabolic activity of NK cells [14, 24]. NK cells displayed increased expression levels of both activation markers (CD69 and CD43) following IL-18 stimulation (Supplementary Fig. 2A). Significantly higher expression levels of TNFR2 protein were observed on NK cells following ex vivo stimulation with IL-2/18, while the expression of TNFR1 was comparable to that of IL-2-stimulated cells as assessed by flow cytometry (Fig. 2B).

To further determine the role of IL-18 signaling in the induction of TNF receptor expression in vivo, we used a mouse model that lacks the *Il18r1* gene, specifically in NK cells [24]. Littermate *Il18r1*^*fl/fl*^ (Cre−) and *NKp46-Cre-Il18r1*^*fl/fl*^ (Cre+) mice were injected with LPS, and splenic NK cells from these mice were analyzed for the expression of TNFRs on D2 post-injection. The expression of TNFR1 on NK cells from Cre+ mice was comparable to that of NK cells from Cre− mice. However, NK cells from Cre+ mice displayed significantly lower expression levels of TNFR2 than NK cells from Cre− mice on D2 post-LPS injection (Fig. 2C). The binding of IL-18 to its receptors triggers an orchestrated signaling pathway that begins with the recruitment of the cytoplasmic adaptor molecule MyD88, which leads to the activation of NF-κB [25]. Consistent with the *Il18r1* mouse model, NK cells from *MyD88*^*−/−*^ mice also failed to upregulate the expression of TNFR2 post-LPS injection (Fig. 2D), demonstrating that IL-18 signaling is critical for the expression of TNFR2 on NK cells in LPS-induced inflammation. Unexpectedly, TNFR2 expression in NK cells was comparable between wild-type and *MyD88*^*−/−*^ mice on D3 post-MCMV infection (Supplementary Fig. 2B). Since the activation of NF-κB can occur in a MyD88-independent manner [26], we further assessed *MyD88-TRIF* double knockout mice challenged with MCMV infection. Similar to *MyD88*^*−/−*^ mice, TNFR2 expression in NK cells from *MyD88-TRIF* double knockout mice was equivalent to that in NK cells from wild-type mice post-MCMV infection (Supplementary Fig. 2C). NK cells from *MyD88*^*−/−*^ and *MyD88-TRIF* double knockout mice also displayed higher expression levels of TNFR1 following MCMV infection, which was not observed in wild-type mice (Supplementary Fig. 2B, C). Taken together, these results demonstrate that signaling through IL-18 can induce TNFR2 expression on NK cells.

### TNFα promotes NK cell proliferation and glycolytic activity

**Fig. 3:**
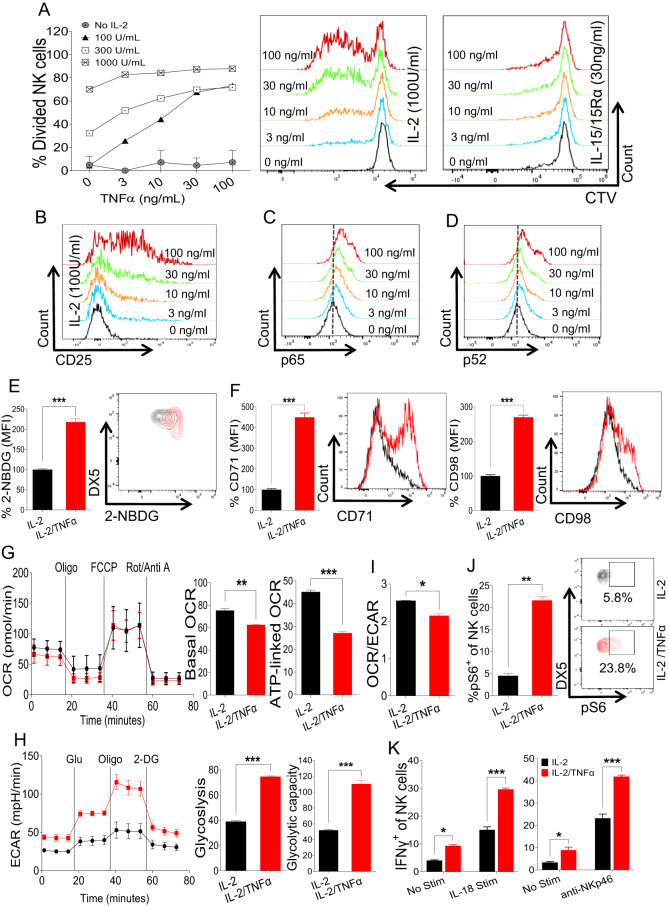
NK cell proliferation and metabolic activity under stimulation with TNFα ex vivo. **A** NK cells were enriched from the spleens of naive C57BL/6 mice and stained with cell trace violet dye followed by stimulation with the indicated doses of rhIL-2, IL-15/IL-15Rα complex, and TNFα ex vivo for 3 days, and the percentage of dividing NK cells was analyzed. **B** CD25 upregulation on NK cells following stimulation with the indicated doses of IL-2 and TNFα. **C–F** NK cells were enriched from the spleens of naive C57BL/6 mice and stimulated with TNFα ex vivo for 48 h. 100 U/ml rhIL-2 was added to maintain NK cell survival. Representative plots depict the expression of (**C**) pNFkB-p65 and (**D**) pNFkB-p52. The graphs represent (**E**) glucose uptake by NK cells as measured by the MFI of 2-NBDG and (**F**) the expression of CD71 and CD98 in cytokine-stimulated NK cells. **G–I** Freshly isolated NK cells were flow-sorted followed by ex vivo TNFα stimulation for 48 h, and the oxygen consumption rate (OCR) and extracellular acidification rate (ECAR) were analyzed. **J** Cells were prepared as in (**C**), and the proportion of pS6^+^ cells among the total NK cell population was calculated. **K** NK cells were enriched from the spleens of naive C57BL/6 mice and treated with TNFα ex vivo for 18 h in the presence of 100 U/ml rhIL-2 followed by stimulation with plate-coated anti-NKp46 or IL-18 for an additional 5 h, and the proportion of IFNγ^+^ cells among the total NK cell population was measured. The MFI expression is presented as a percentage relative to the MFI of control cells as 100. Data are from one experiment representative of three independent experiments, with at least two replicates per group. Data represent the mean ± SD. *p < 0.05; **p < 0.01; ***p < 0.001

Since proliferation often coincides with NK cell activation [10, 27], we investigated the effects of TNFα signaling on NK cell proliferation and metabolic activity ex vivo. Freshly enriched NK cells were stimulated with different doses of recombinant human (rh) IL-2 and murine TNFα, and NK cell division was measured by a cell trace violet dilution assay. Following TNFα stimulation, increased numbers of divided NK cells were observed in the presence of IL-2 (Fig. 3A). Interestingly, a dose-dependent effect was predominantly observed in the presence of low dose of IL-2 (100 U/ml), whereas increasing the IL-2 concentration (300 U/ml) lessened the TNFα effect, and this effect was negligible at the highest concentration of IL-2 tested (1000 U/ml) (Fig. 3A). This observation suggested that TNFα might increase the responsiveness of NK cells toward low doses of IL-2. We next examined the expression of IL-2 receptor alpha chain (IL‐2Rα, CD25), which plays a critical role in NK cell responsiveness to low doses of IL-2. In accordance with the enhanced proliferation, NK cells displayed increased expression levels of CD25 in a dose-dependent manner following TNFα stimulation (Fig. 3B; Supplementary Fig. 3A). Notably, TNFα stimulation did not induce NK cell proliferation in the presence of IL-15, suggesting that TNFα-induced NK cell proliferation is mediated by CD25 upregulation by TNFα. (Fig. 3A). Following TNFα stimulation, NK cells also displayed higher expression levels of the cellular proliferation marker Ki-67 (Supplementary Fig. 3B) and activation markers (Supplementary Fig. 3C). Since TNFα is known to induce CD25 expression via NF-κB signaling [28–33], we measured the expression of phospho-NF-κB (pNF-κB) in NK cells stimulated with different concentrations of TNFα. In accordance with CD25 expression, following ex vivo TNFα stimulation, NK cells upregulated the expression of pNF-κB in a dose-dependent manner (Fig. 3C, D). Altogether, these results demonstrate that TNFα signaling enhances NK cell proliferation by upregulating CD25 expression.

Proliferating NK cells require high energy levels for the biosynthesis of diverse cellular compartments. The elevated metabolic demand of NK cells required for enhanced proliferation is fulfilled by increasing the expression levels of nutrient transporters to enable increased nutrient uptake [24]. First, we assessed forward scatter as an indicator of cell size, with high levels being associated with proliferative and metabolically active NK cells. Consistently, we observed an increase in NK cell size upon TNFα stimulation (Supplementary Fig. 3D). To assess the metabolic activity of NK cells and to evaluate the expression of glucose transporters, we analyzed the glucose uptake capacity of NK cells after TNFα stimulation by using 2-NBDG, a fluorescent glucose analog. Following TNFα stimulation, NK cells displayed higher 2-NBDG uptake, suggesting upregulated glucose transporter expression (Fig. 3E). The expression of transferrin receptor (CD71) and amino acid transporter (CD98) was also significantly upregulated in the TNFα-stimulated NK cell population (Fig. 3F).

To further probe the metabolic profile of NK cells in regard to their cellular energetic state, we measured the oxygen consumption rate (OCR) and extracellular acidification rate (ECAR) of NK cells following TNFα stimulation. Interestingly, NK cells, when treated with TNFα, exhibited decreased basal OCR and significantly reduced ATP-linked respiration (Fig. 3G). Conversely, TNFα stimulation enhanced glycolysis and the glycolytic capacity when cells were subjected to a glycolytic stress test compared to IL-2 stimulation (Fig. 3H). These findings indicate that signaling through TNFα skews NK cell metabolism from oxidative phosphorylation to aerobic glycolysis, as evidenced by the OCR/ECAR ratio (Fig. 3I). Increased mTORC1 activity measured by the expression of pS6, a master regulator of aerobic glycolysis, further supported this notion (Fig. 3J). Finally, we determined whether TNFα-induced metabolic changes enhance NK cell function. We observed an increased number of granzyme B^+^ cells among total NK cells after TNFα stimulation (Supplementary Fig. 3E). Furthermore, TNFα signaling resulted in augmented IFN-γ production and degranulation when NK cells were stimulated with either anti-NKp46 or IL-18 (Fig. 3K; Supplementary Fig. 3F). Overall, these results demonstrate that TNFα can induce enhanced proliferation, elevated expression levels of nutrient transporters, and metabolic shifting to aerobic glycolysis in NK cells, thereby enhancing their effector functions.

### IL-18 augments TNFα-induced NK cell proliferation and function

**Fig. 4:**
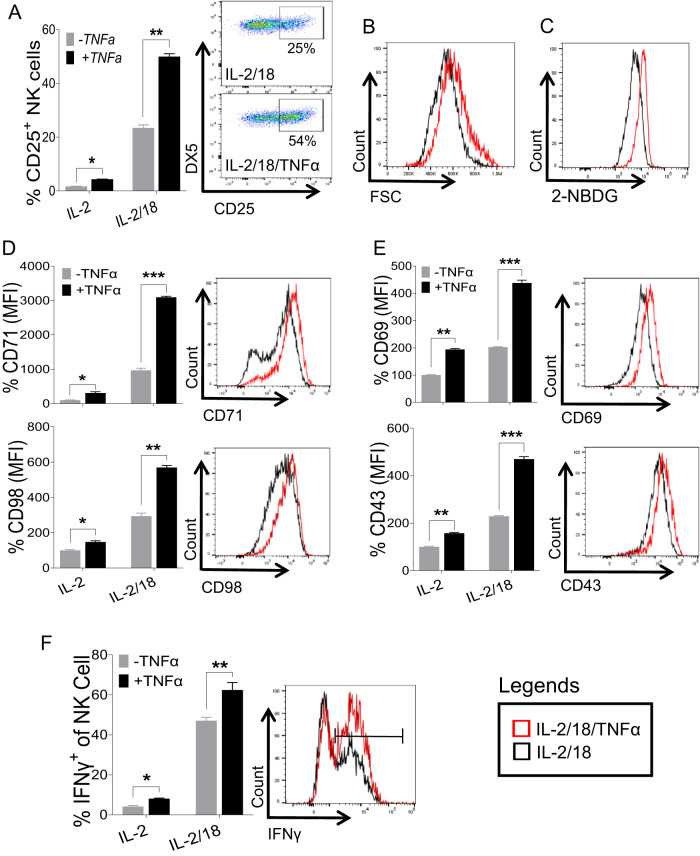
IL-18/TNFα synergistically augments NK cell activation and metabolic activity. NK cells were enriched from the spleens of naive C57BL/6 mice and stimulated with IL-18 (20 ng/ml) in the presence or absence of TNFα (30 ng/ml) ex vivo. 100 U/ml rhIL-2 was added to maintain NK cell survival. **A** Representative graphs depict the proportion of CD25^+^ cells among the total NK cell population after 24 h. **B** Histogram of cell size as measured by FSC, (**C**) glucose uptake by NK cells as measured by 2-NBDG, (**D**) expression of CD71 and CD98, and (**E**) expression of CD69 and CD43 following the stimulation of NK cells with IL-2/18 (black) and IL-2/18/TNFα (red) for 48 h are shown. **F** Enriched NK cells from the spleens of naive C57BL/6 mice were stimulated with IL-18 in the presence or absence of TNFα ex vivo for 24 h. 100 U/ml rhIL-2 was added to maintain NK cell survival. The graph represents the proportion of IFNγ^+^ cells among the total NK cell population. The MFI expression is presented as a percentage relative to the MFI of control cells as 100. Data are from one experiment representative of three independent experiments, with at least two replicates per group. Data represent the mean ± SD. *p < 0.05; **p < 0.01; ***p < 0.001

The observation that IL-18 signaling upregulated the expression of TNFRs on NK cells (Fig. 2A, B) prompted us to assess whether IL-18 can enhance the TNFα-induced activation of NK cells. First, we analyzed the induction of CD25 since IL-18 priming was shown to upregulate CD25 expression on NK cells, thereby increasing NK cell sensitivity to IL‐2 [18]. Surprisingly, the addition of TNFα drastically increased the number of IL-18-induced CD25^+^ cells among total NK cells (Fig. 4A) when freshly isolated NK cells were stimulated with IL-18 in the presence of TNFα ex vivo. Considering the upregulated expression of CD25 on NK cells following IL-18/TNFα treatment, we next assessed the metabolic changes in these NK cells. Compared to NK cells stimulated with IL-2/18 alone, NK cells stimulated with TNFα exhibited increased cell size (Fig. 4B) and glucose uptake (Fig. 4C). Similarly, NK cells with combined stimulation of IL-2/18/TNFα displayed significantly higher expression levels of CD71 and CD98 (Fig. 4D), suggesting that the combination of IL-18/TNFα has a synergistic effect on the metabolic activity of NK cells. In addition, NK cells displayed significantly higher expression levels of CD69 and CD43 on NK cells when stimulated with combined IL-2/18 and TNFα (Fig. 4E). Moreover, we observed significantly higher IFN-γ production in IL-18-stimulated NK cells in the presence of TNFα (Fig. 4F). Altogether, these data demonstrate that IL-18 amplifies TNFα-induced activation of NK cells presumably by increasing the responsiveness toward TNFα through the higher expression levels of TNFRs on the cell surface.

### Autocrine TNFα is required for optimal proliferation and glycolytic activity in NK cells

**Fig. 5:**
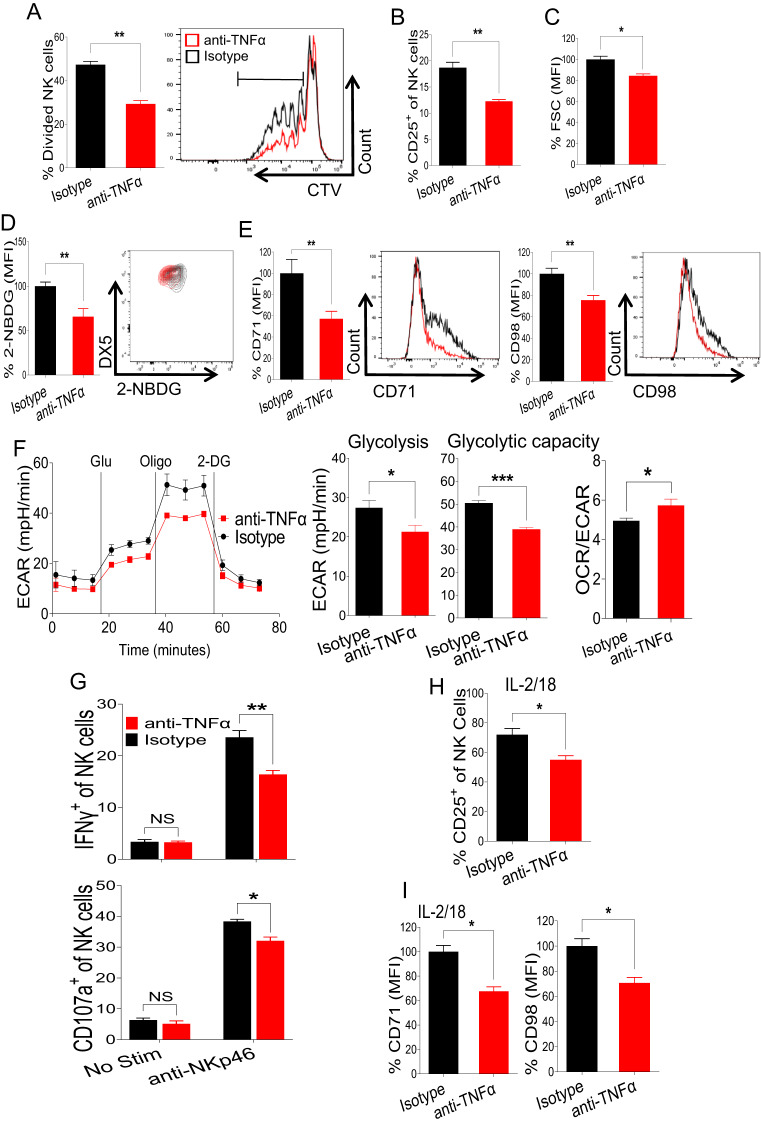
Autocrine TNFα is critical for the proliferation and glycolytic activity of NK cells. **A** NK cells were enriched from the spleens of naive C57BL/6 mice and stained with cell trace violet dye followed by the addition of isotype control or anti-TNFα antibody in the presence of 100 U/ml rhIL-2 in the media. The percentage of dividing NK cells was assessed on Day 4 post-treatment. **B–E** Enriched NK cells from the spleens of naive C57BL/6 mice were treated with isotype control or anti-TNFα antibody in the presence of 100 U/ml rhIL-2, and the indicated parameters were analyzed after 40 h of stimulation. **B** Representative graph indicates the proportion of CD25^+^ cells among the total NK cell population of antibody-treated cells. The MFI of (**C**) FSC in NK cells indicating cell size, (**D**) glucose uptake by NK cells as measured by 2-NBDG, and (**E**) CD71 and CD98 expression of antibody-treated NK cells are presented. **F** Freshly isolated NK cells from the spleens of naive C57BL/6 mice were flow-sorted followed by treatment with isotype control or anti-TNFα antibody in the presence of 100 U/ml rhIL-2, and a glycolysis stress test was performed in glucose-free media to analyze the extracellular acidification rate. **G** NK cells were enriched from the spleens of naive C57BL/6 mice and treated with isotype control or anti-TNFα antibody in the presence of 100 U/ml rhIL-2 ex vivo for 18 h followed by stimulation with plate-coated anti-NKp46 for an additional 5 h, and the proportion of IFNγ^+^ and CD107a^+^ cells among the total NK cell population was measured. Enriched NK cells from the spleens of naive C57BL/6 mice were treated with isotype control or anti-TNFα antibody in the presence of 100 U/ml rhIL-2 and IL-18 (20 ng/ml) for 40 h. Representative graphs demonstrate (**H**) the proportion of CD25^+^ cells among the total NK cell population and (**I**) the MFI of CD71 and CD98 expression of antibody-treated NK cells. The MFI expression is presented as a percentage relative to the MFI of control cells as 100. Data are from one experiment representative of two independent experiments, with at least three replicates per group. Data represent the mean ± SD. *p < 0.05; **p < 0.01; ***p < 0.001; ns nonsignificant

TNFα is produced by a variety of immune cells, including NK cells [3–5]. To investigate the effect of autocrine TNFα signaling on NK cell proliferation and metabolic activity, freshly enriched NK cells were treated with isotype control or anti-TNFα antibodies, and NK cell proliferation was assessed in the presence of 100 U/ml rhIL-2 ex vivo. We observed that treatment with the TNFα neutralizing antibody significantly reduced the proliferation of NK cells (Fig. 5A). Interestingly, decreased NK cell proliferation correlated with reduced expression levels of CD25 on NK cells (Fig. 5B). We reasoned that blocking autocrine TNFα signaling would impact NK cell metabolic activity. Anti-TNFα antibody treatment decreased NK cell size as measured by forward scatter (Fig. 5C). In agreement with this decrease in cell size, treatment with anti-TNFα antibody resulted in decreased glucose uptake (Fig. 5D) and reduced expression levels of CD71 and CD98 on NK cells compared to those of control cells (Fig. 5E), indicating an overall suppression of cellular metabolic activity by blocking autocrine TNFα signaling.

Considering that TNFα signaling promoted aerobic glycolysis in NK cells (Fig. 3G, H), we tested whether blocking intrinsic TNFα signaling has any consequences on the metabolic pathways in NK cells. Freshly sorted NK cells were treated with isotype control or anti-TNFα antibodies, and the glycolysis stress test was performed. Notably, blocking autocrine TNFα signaling decreased both basal glycolysis and the cellular glycolytic capacity of NK cells, while cells displayed an increased OCR/ECAR ratio (Fig. 5F). However, the mitochondrial stress test analysis did not show any impact on cellular respiration following anti-TNFα antibody treatment (Supplementary Fig. 4A), further supporting the role of TNFα signaling in NK cell glycolysis. Additionally, NK cells treated with anti-TNFα antibodies displayed significantly lower expression levels of the activation marker CD69 (Supplementary Fig. 4B). In agreement with reduced proliferation and metabolic activity, anti-TNFα-treated cells also exhibited decreased numbers of IFN-γ^+^ and CD107a^+^ cells among total NK cells compared to those exhibited by isotype-treated cells following stimulation with anti-NKp46 (Fig. 5G).

We next determined whether autocrine TNFα signaling is important for the IL-18-induced biological function of NK cells. Notably, blocking autocrine TNFα downregulated the IL-18-induced expression of CD25 (Fig. 5H) and nutrient transporters (Fig. 5I) in NK cells. Taken together, these results demonstrate that autocrine TNFα is critical for the optimal proliferation, expression of nutrient transporters, and glycolytic metabolism of NK cells.

### TNFR2 is essential for TNFα-induced NK cell proliferation and metabolic activity

Signaling of TNFα is complex and occurs through two receptors, TNFR1 and TNFR2, in a context-dependent manner [2, 7]. Interestingly, NK cells stimulated with IL-2 and IL-18 showed decreased expression levels of both receptors upon TNFα addition, suggesting that both receptors engage with TNFα (data not shown). To dissect the TNFα-induced activation of NK cells in regard to the respective receptor, we decided to take advantage of mouse models deficient in either *Tnfrsf1a* (TNFR1 KO) or *Tnfrsf1b* (TNFR2 KO). Notably, TNFR1 and TNFR2 expression on NK cells was not affected in mice deficient in the other counterpart receptor, suggesting that the surface expression of each TNF receptor is independently regulated (Supplementary Fig. 5A). Deficiency of TNFR1 or TNFR2 did not affect the proportion and number of immune cells at steady state, as the frequencies of lymphoid immune cells were comparable in the spleens of wild-type, TNFR1 KO, and TNFR2 KO mice (Supplementary Fig. 5B). Furthermore, the maturation of NK cells was also comparable in the bone marrow, spleens and livers of wild-type, TNFR1 KO, and TNFR2 KO mice (Supplementary Fig. 5C, D), suggesting that TNFα signaling is dispensable during the development of NK cells at steady state. Since no aberrant development in NK cells was observed in TNFR1 KO and TNFR2 KO mice, we reasoned that NK cells from the mice deficient in either TNFR1 or TNFR2 are advantageous to determine the role of TNFα/TNFRs signaling on NK cell proliferation.

**Fig. 6:**
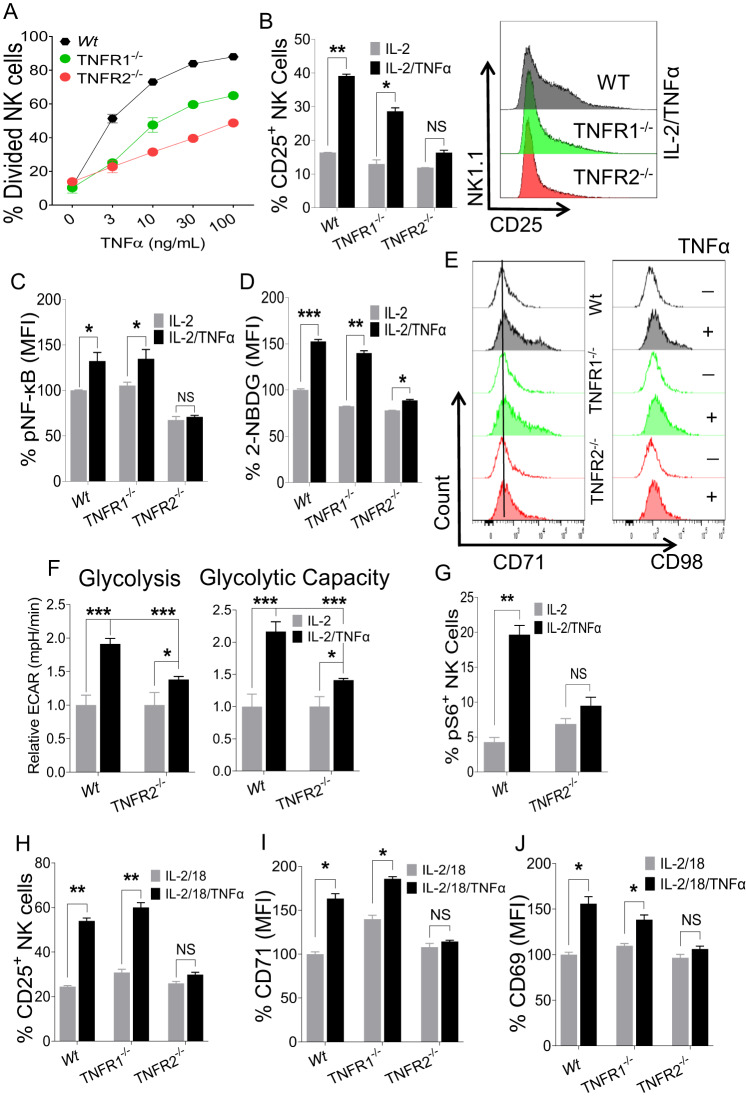
The effect of TNFα on NK cells from mice deficient in either TNFR1 or TNFR2. **A** NK cells were enriched from the spleens of the naive indicated mice and stained with cell trace violet dye followed by stimulation with the indicated doses of TNFα ex vivo for 3 days, and the percentage of dividing NK cells was analyzed. 100 U/ml rhIL-2 was added to maintain NK cell survival. **B–E** Enriched splenic NK cells from the indicated mice were stimulated with TNFα (30 ng/ml) ex vivo for 48 h in the presence of 100 U/ml rhIL-2. **B** The proportion of CD25^+^ cells among the total NK cell population of cytokine-stimulated cells. The graphs indicate the MFI of (**C**) pNF-κB-p65, (**D**) glucose uptake as measured by 2-NBDG and (**E**) representative plots of CD71 and CD98 expression on cytokine-stimulated cells. **F** Freshly isolated NK cells from the spleens of the naive indicated mice were flow-sorted followed by stimulation with TNFα ex vivo in the presence of 100 U/ml rhIL-2, and a glycolysis stress test was performed in glucose-free medium to analyze the relative extracellular acidification rate. **G** Cells were prepared as in (**B**), and the proportion of pS6^+^ cells among the total NK cell population was calculated. Enriched NK cells from the spleens of the indicated naive mice were stimulated ex vivo with IL-18 in the presence or absence of TNFα (30 ng/ml) for 48 h. 100 U/ml rhIL-2 was added to maintain NK cell survival. Graphs demonstrate (**H**) the proportion of CD25^+^ cells among the total NK cell population and the MFI of (**I**) CD71 and (**J**) CD69 expression on cytokine-stimulated NK cells. The MFI expression is presented as a percentage relative to the MFI of control cells as 100. Data are from one experiment representative of at least two independent experiments, with at least three replicates per group. Data represent the mean ± SD. *p < 0.05; **p < 0.01; ***p < 0.001; ns nonsignificant

Freshly enriched NK cells from wild-type, TNFR1 KO, and TNFR2 KO mice were treated with different doses of TNFα in the presence of IL-2 (100 U/ml), and NK cell proliferation was assessed. NK cells from TNFR2 KO mice showed significantly lower cell division following TNFα stimulation (Fig. 6A). NK cells from TNFR1 KO mice also displayed decreased proliferation compared to that of NK cells from wild-type mice but their proliferation was still considerably higher than that of TNFR2 KO mice (Fig. 6A). A similar observation was made when the expression of Ki-67 on NK cells was assessed (Supplementary Fig. 6A). Interestingly, NK cells from TNFR2 KO mice failed to upregulate CD25 expression following TNFα stimulation (Fig. 6B). Importantly, TNFα stimulation did not induce pNF-κB expression in NK cells from TNFR2 KO mice (Fig. 6C), indicating that TNFR2 mediates TNFα-induced NF-κB activation. Next, we assessed the metabolic activity of NK cells from all groups. NK cells from TNFR2 KO mice displayed downregulated expression of glucose transporters in terms of glucose uptake (Fig. 6D) and nutrient transporters following TNFα stimulation (Fig. 6E; Supplementary Fig. 6B) compared to those from wild-type and TNFR1 KO mice ex vivo. In particular, NK cells from TNFR2 KO mice displayed significantly lower glycolysis and cellular glycolytic capacity when subjected to the glycolysis stress test (Fig. 6F) than NK cells from wild-type mice. In a similar manner, NK cells from TNFR2 KO mice were unable to induce mTORC1 activity following TNFα stimulation, further signifying reduced glycolytic activity in these cells (Fig. 6G). In accordance with reduced metabolic activity, compared to NK cells from wild-type mice stimulated with anti-NKp46, NK cells from TNFR2 KO mice displayed decreased IFN-γ production and degranulation (Supplementary Fig. 6C). Notably, NK cells from TNFR2 KO mice produced significantly less TNFα than those from wild-type mice (Supplementary Fig. 6C). Furthermore, NK cells from TNFR2 KO mice displayed reduced production of granzyme B (Supplementary Fig. 6D) and failed to induce CD69 expression (Supplementary Fig. 6E) following TNFα stimulation.

Finally, to evaluate the role of the TNFα/TNFR2 axis in the IL-18-induced activation of NK cells, freshly isolated NK cells were stimulated with IL-18 in the presence of TNFα ex vivo. We did not observe CD25 induction in NK cells from TNFR2 KO mice in the presence of TNFα (Fig. 6H). A similar observation was made when the expression of CD71 (Fig. 6I) and CD69 (Fig. 6J) was analyzed. However, the expression of CD98 and CD43 did not differ on NK cells from wild-type mice and TNFR2 KO mice (data not shown) following TNFα stimulation. Collectively, our data demonstrate that the TNFα/TNFR2 axis is critical in regulating the proliferation and metabolic activity of NK cells, and hampering this axis impairs NK cell proliferation, leading to reduced metabolic activity.

### TNFR2 expression is critical for the gene expression profiles associated with NK cell metabolic reprogramming during virus infection

**Fig. 7:**
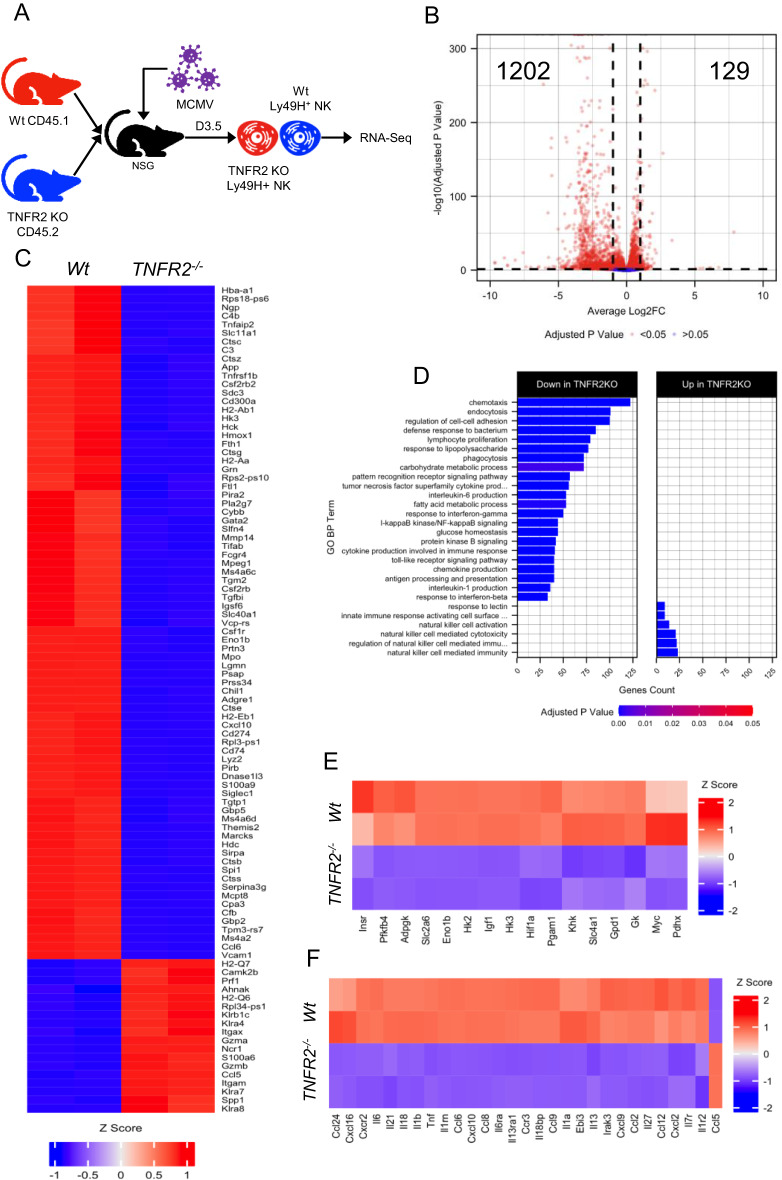
NK cells from TNFR2 KO mice displayed reduced expression levels of the genes associated with glycolytic metabolic programming following acute MCMV infection in vivo. **A** Purified NK cells from wild-type (CD45.1) and TNFR2 KO (CD45.2) mice were co-transferred  into NSG mice. One day later, mice were intraperitoneally infected with MCMV. Mice were sacrificed on D3.5 following infection, and splenic Ly49H^+^CD45.1^+^ and Ly49H^+^CD45.2^+^ NK cells were sorted by flow cytometry and used for bulk RNA sequencing (RNA-seq) analysis. **B** Volcano plot depicting differentially expressed genes (adjusted *p* value < 0.05 and 1 log2-fold change cutoff) between wild-type and TNFR2 KO NK cells. **C** Heatmap and hierarchical clustering of the top 100 differentially expressed genes, ranked by adjusted *p* value. **D** Over-representation analysis of Gene Ontology Biological Process (GO BP) on significantly (adjusted *p* value < 0.05) differentially expressed genes with upregulated (log2-fold change > 0.5) and downregulated (log2-fold change < −0.5) expression in NK cells from wild-type and TNFR2 KO mice. Heatmap and hierarchical clustering of selected differentially expressed genes involved in aerobic glycolysis (**E**) and cytokines, chemokines, and their receptors (**F**) in NK cells from wild-type versus TNFR2 KO mice. Data are from one experiment performed in duplicate, and samples were isolated from the pooled spleen sample generated from three infected mice

To more comprehensively characterize the role of TNFR2 signaling in NK cells, we performed transcriptomic analysis of TNFR2-deficient NK cells on D3.5 following MCMV infection. To employ an unbiased approach, purified NK cells from wild-type (CD45.1) and TNFR2 (CD45.2) were co-transferred into NSG mice, followed by MCMV infection. Ly49H^+^CD45.1^+^, and Ly49H^+^CD45.2^+^ NK cells were sorted by flow cytometry and used for bulk RNA sequencing (RNA-seq) analysis (Fig. 7A). We identified 1331 differentially expressed genes with an adjusted *p* value under 0.05 and an absolute log2-fold change greater than 1 (Fig. 7B). Of these differentially expressed genes, 1202 were downregulated and only 129 were upregulated in NK cells from TNFR2 KO mice (Fig. 7B). The heatmap of the top 100 genes ranked by adjusted *p* value is shown in Fig. 7C. Gene ontology over-representation analysis revealed that multiple biological processes are compromised in TNFR2-deficient NK cells, including pathways involved in cellular metabolism, proliferation and cytokine production (Fig. 7D). Next, we focused on genes involved in glycolysis and found significantly reduced expression levels of several genes in TNFR2-deficient NK cells (Fig. 7E). Notably, we observed a strong decrease in the expression levels of genes involved in the first step of glucose metabolism pathways (HK2, HK3), further confirming the role of TNFR2 signaling in NK cell glycolysis. Beyond glycolysis, we observed broad dysfunction in other metabolic processes in NK cells from TNFR2 KO mice, as indicated by the reduced expression levels of other metabolism-related genes (Supplementary Fig. 7A). Importantly, we also found reduced expression levels of the genes involved in cytokine production (Fig. 7F), suggesting that TNFR2 signaling is critical for NK cell-induced adaptive immune responses. Similar to the lower TNFα expression level (Supplementary Fig. 6C), the reduced expression levels of numerous genes involved in TNFα signaling in NK cells from TNFR2 KO mice further strengthens the predominant role of the autocrine mechanism in NK cells (Supplementary Fig. 7B). Altogether, our transcriptomic analysis revealed that TNFR2 signaling is crucial for the metabolic reprogramming of NK cells during viral infection.

### TNFR2 is indispensable for optimum NK cell expansion and function during MCMV infection in vivo

**Fig. 8:**
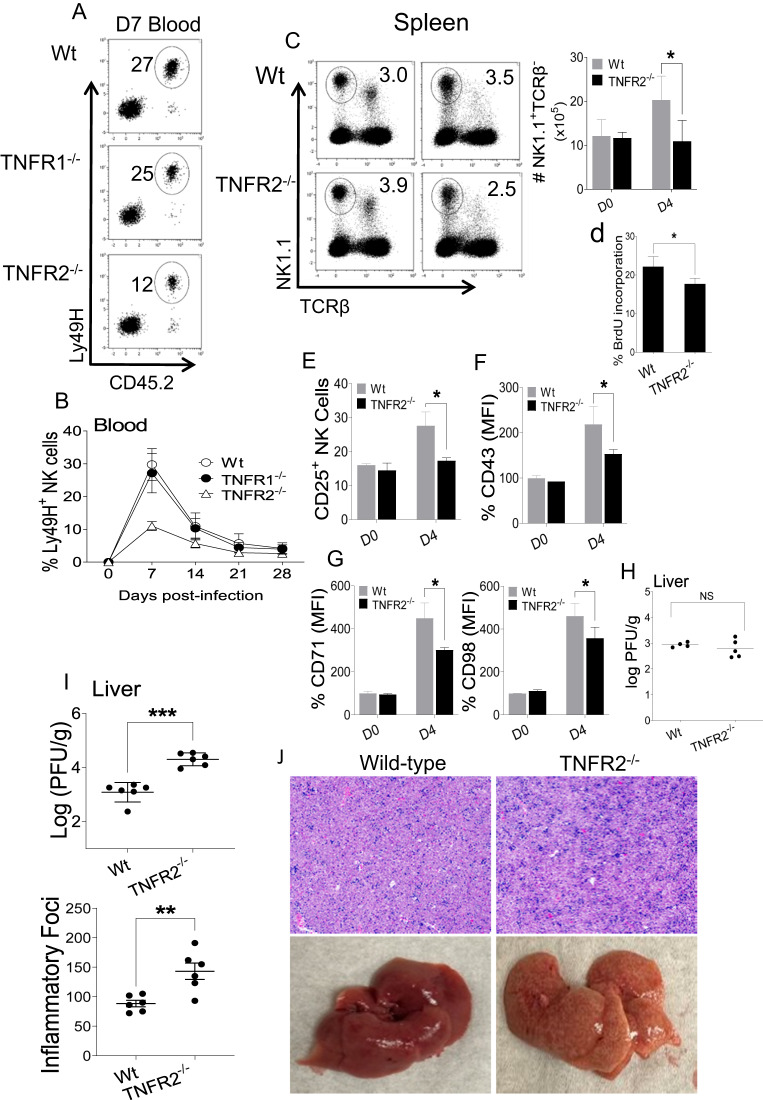
Intrinsic signaling through TNFR2 for NK cell proliferation during acute MCMV infection in vivo. Splenic NK cells of C57BL/6, TNFR1^−/−^, and TNFR2^−/−^ mice (CD45.2^+^) were isolated and transferred into Ly49H^−/−^ recipient (CD45.1^+^) mice. Ly49H^−/−^ mice were either uninfected or intraperitoneally given 3000 PFU MCMV one day following adoptive transfer. Peripheral leukocytes in the blood of recipient mice were harvested and analyzed every 7^th^ day post-infection. **A** Representative plots show transferred cells (Ly49H^+^CD45.2^+^) gated on DX5^+^TCRβ^-^ cells in the blood of recipient mice on Day 7. **B** Representative graph of transferred cells (Ly49H^+^CD45.2^+^) gated on DX5^+^TCRβ^-^ cells from the blood of recipient mice on the indicated days post-infection. C57BL/6 and TNFR2^−/−^ mice were either uninfected or intraperitoneally challenged with 3000 PFU MCMV, and splenic leukocytes were analyzed from naive (D0) or infected mice on Day 4 (D4) post-infection to assess (**C**) NK cell proportion and (**D**) BrdU incorporation of NK cells from the spleens of the indicated mice. Representative graphs represent the (**E**) proportion of CD25^+^ cells among the total NK cell population, (**F**) MFI of CD43, (**G**) CD71 and CD98 expression in NK cells from indicated mice, and (**H**) viral titers in the liver of indicated mice. Equal numbers (50,000) of purified splenic Ly49H^+^ NK cells from wild-type and TNFR2^−/−^ mice were transferred into *Rag2*^−/−^*Il2rg*^−/−^ mice. Mice were intraperitoneally infected with MCMV one day following adoptive transfer. On Day 7 post-infection, livers were collected to assess (**I**) viral titer and (**J**) inflammatory foci through H&E staining. Data are from one experiment representative of two independent experiments, with at least 4 mice per group. The MFI expression is presented as a percentage relative to the MFI of control mice as 100. Data represent the mean ± SD. *p < 0.05; **p < 0.01; ***p < 0.001; ns nonsignificant

RNA-seq data revealed an overall attenuation of the metabolic and functional activity of TNFR2 KO NK cells. First, to delineate the intrinsic effect of TNFR2 on NK cell proliferation in vivo, an adoptive transfer experiment was carried out using splenic NK cells from wild-type, TNFR1 KO or TNFR2 KO mice to evaluate Ly49H-induced proliferation during MCMV infection. We adoptively transferred equal numbers of Ly49H^+^ NK cells from either wild-type, TNFR1 KO or TNFR2 KO (CD45.2^+^) mice into Ly49H-deficient recipient (CD45.1^+^) mice. One day later, recipient mice were infected with MCMV. Mice were bled every 7 days post-infection until Day 28, and the NK cell population was analyzed by gating on Ly49H^+^CD45.2^+^ cells. Notably, NK cells from TNFR2 KO mice demonstrated a significant decrease in Ly49H-induced proliferation during MCMV infection compared to those from wild-type and TNFR1 KO mice on D7 postinfection (Fig. 8A). Accordingly, NK cells from TNFR2 KO mice displayed decreased expansion over time (Fig. 8B), indicating that TNFR2 is required for Ly49H^+^ NK cell expansion following MCMV infection. These results demonstrated that intrinsic signaling through TNFR2 in NK cells is critical for NK cell proliferation during acute MCMV infection in vivo.

Next, to understand the importance of the TNFα/TNFR2 axis in the antiviral activity of NK cells in virus infection, we compared cell proportions, activation, and metabolic states of NK cells from wild-type and TNFR2 KO mice on D4 post-MCMV infection. Consistent with the data from the adoptive transfer experiment (Fig. 8A, B), the proportions and number of splenic NK cells from TNFR2 KO mice were considerably lower than those of wild-type mice on D4 post-infection (Fig. 8C). Likewise, splenic NK cells from TNFR2 KO mice displayed decreased BrdU incorporation compared to those from wild-type mice (Fig. 8D). Furthermore, NK cells from TNFR2 KO mice exhibited decreased activation, as evidenced by lower expression of CD25 (Fig. 8E) and CD43 (Fig. 8F) in NK cells. Similarly, these NK cells demonstrated impaired metabolic activity in terms of nutrient transporter expression (Fig. 8G) compared to that of the NK cells from control mice. However, we did not observe any difference in viral load between these mice (Fig. 8H). Finally, we examined viral burdens and liver pathology in immune-deficient *Rag2*^*−/*−^*Il2rg*^*−/*−^ mice that received NK cells from either wild-type or TNFR2 KO mice followed by MCMV infection. Notably, the adoptively transferred NK cells from TNFR2 KO mice provided less protection than those from wild-type mice, as indicated by higher viral titers (Fig. 8I) and increased inflammation quantified by the numbers of inflammatory foci (Fig. 8J) in the livers of infected mice on Day 7 post-MCMV infection. Collectively, these findings demonstrate the critical role of the TNFα/TNFR2 axis in regulating the proliferation and metabolic activity of NK cells during MCMV infection, thereby contributing to efficient antiviral responses.

## Discussion

TNFα, a highly pleiotropic cytokine, plays a crucial role in regulating the balance between activation and death in various cell types through its two receptors, TNFR1 and TNFR2 [3–5]. Accumulated evidence has demonstrated that TNFR1 is ubiquitously expressed in almost all cells, while TNFR2 exhibits limited expression in immune cells such as T cells, Treg cells, and dendritic cells. Overall, signaling through TNFR1 leads to the activation of proinflammatory and apoptotic pathways; however, TNFR2 has no death domain and can promote cell proliferation [34, 35]. Therefore, the pleiotropic effects of TNFα can be explained by the crosstalk of soluble and membrane-bound TNFα and the two TNF receptors, TNFR1 and TNFR2, in a cell-specific and context-dependent manner. Although TNFα expression is considered a pro-inflammatory cytokine commonly observed in early virus infection in which NK cells are highly activated by pro-inflammatory cytokines [15, 16, 18], the immunomodulatory nature of TNFα’s effect on NK cells in the context of TNFR1 and TNFR2 during virus infection remains largely elusive. A report showed that crosstalk between dendritic cells (DCs) and NK cells through the TNFα-TNFR2 axis is required for DC-mediated activation of NK cell proliferation and cytotoxic activity [20]. Since TNF is also considered a metabolic messenger [36], an enormous interest in immunometabolism focuses on linking metabolism to immune cell function. Thus, investigating the role of TNFα in NK cell metabolism is critical for a better understanding of its role in immunoregulation.

Here, we identified that TNFα-TNFR2 signaling is a major mechanism by which NK cells enhance proliferation by glycolytic metabolism. During MCMV- and LPS-induced inflammation, TNFR2 expression is highly induced and further enhanced by IL-18 stimulation. Notably, in LPS-induced inflammation, TNFR2 induction largely depended on the IL-18-MyD88 pathway (Fig. 2C, D). TNFα treatment enhanced NK cell proliferation, predominantly through CD25 induction on NK cells, rendering them highly sensitive to lower concentrations of IL-2 (Figs. 3, 4). Metabolically, TNFα treatment increased glucose uptake and the expression levels of nutrient transporters, and the effect was further enhanced by coincubation with IL-18. Seahorse metabolic profiling indicated that TNFα treatment switched the metabolic balance to aerobic glycolysis (Fig. 3). Interestingly, blocking autocrine TNFα affected NK cell proliferation and metabolic activity in isolated NK cell cultures, suggesting the auto/paracrine effect of the cytokine (Fig. 5). Furthermore, mechanistic studies using NK cells deficient in either TNFR1 or TNFR2 allowed us to demonstrate the critical role of TNFR2 signaling in proliferation and nutrient transporter expression (Fig. 6). RNA-seq analysis of NK cells further validated that TNFR2 expression is critical for NK cell metabolic reprogramming to the glycolytic pathway during virus infection at the transcriptional level. Most of the genes associated with glycolysis were reduced in NK cells deficient in TNFR2 during MCMV infection (Fig. 7). Finally, an adoptive transfer experiment clearly demonstrated that TNFR2 is indispensable for the proper expansion of Ly49H^+^ NK cells, leading to effective virus control upon MCMV infection (Fig. 8). Notably, most experiments were performed with highly purified NK cells (>80% by magnetic separation and >95% by cell sorting) in this manuscript. Thus, our discoveries were obtained in the absence of dendritic cells and should be different from those occurring in the interaction with dendritic cells [20].

We identified MyD88-dependent TNFR2 expression in NK cells induced by LPS-induced inflammation but not by MCMV infection (Fig. 2 and Supplementary Fig. 2). This discrepancy might be explained by the complex crosstalk of multiple signaling pathways from cytokine and/or activating receptors during MCMV infection [15, 16, 18]. In addition, MCMV susceptibility is highly increased in mice deficient in *MyD88* or *Trif* [37–40]. Thus, excessive inflammation associated with increased MCMV susceptibility might induce TNFR2 upregulation on NK cells from cytokines and/or infected cells, which may compensate for IL-18-MyD88 signaling. Interestingly, NK cells from *MyD88*^*−/−*^ and *MyD88-TRIF* double knockout mice showed higher expression levels of TNFR1 following MCMV infection (Supplementary Fig. 2). In addition, the defect in Ly49H-mediated preferential expansion of NK cells deficient in TNFR2 was rather unexpected because Ly49H-mediated expansion was shown to be intact in mice deficient in TNFα [41]. This report concluded that IL-12 and IL-18 are required for the expansion of Ly49H^+^ NK cells during MCMV infection. However, these results were not reproduced in another paper demonstrating that Ly49H^+^ NK cell expansion is intact in mice deficient in IL-12, IL-18, and IL-18R [22]. Altogether, these results suggest compensatory crosstalk from multiple signaling pathways involving cytokines and activating receptors in the activation of NK cells during MCMV infection.

Although it has been shown that soluble TNFα has a higher affinity for TNFR1, while membrane-bound TNFα favors TNFR2 [42, 43], we observed that soluble TNFα could bind to both TNFR1 and TNFR2. High expression of TNFR2 is one of the signature phenotypes of Treg cells [44–46], and TNFα-TNFR2 signaling is well characterized in Treg cells [47]. TNFα-TNFR2 signaling plays a crucial role in the expansion, suppressive function, and identity of Treg cells, making TNFR2 a prime clinical target for autoimmune diseases [48]. Both thymic and peripheral murine CD4^+^CD25^+^ Tregs express remarkably high levels of TNFR2 relative to CD4^+^CD25^−^ effector T cells, suggesting a link between CD25 and TNFR2 expression [49, 50]. Moreover, TNFα was identified as a mechanistic link between inflammation and metabolism by inducing insulin resistance [51] and metabolic effects on immune cells. For example, a recent study highlighted that human thymus-derived Treg (tTreg) cells become glycolytic and increasingly proliferate in response to TNFR2 costimulation [52], suggesting that tTreg and NK cells may share similar TNFα-TNFR2 biology in proliferation and metabolism.

It was interesting to observe the dramatic increases in viral loads and during the adoptive transfer experiments upon MCMV infection (Fig. 8I, J), as there was no significant difference in MCMV replication between wild-type and TNFR2 KO mice (Fig. 8H). The discrepancy can be attributed to the need for extensive proliferation of NK cells to efficiently control virus replication. Even though both models induce NK cell proliferation, the extent to which NK cells proliferate is far different. In wild-type mice, during MCMV infection, Ly49H^+^ NK cells have been shown to undergo substantial clonal expansion, resulting in several-fold increases in cell numbers. [19, 53]. In contrast, studies have demonstrated that Ly49H^+^ NK cells adoptively transferred into immune-deficient *Rag2*^*−/−*^*Il2rg*^*−/−*^ mice can undergo over 1,000-fold expansion on average in this model [54, 55], although the number of divisions of Ly49H^+^ NK cells in the adoptive transfer experiment can vary depending on several factors, such as the initial number of Ly49H^+^ NK cells transferred, the viral load, the duration of the infection, and the kinetics of NK cell proliferation. Based on our findings, we reason that the lack of significant differences in viral loads between wild-type and TNFR2 KO mice may be due to the sufficient basal number of NK cells in TNFR2 KO mice to control viral replication without the need for extensive proliferation. However, in immunocompromised mice that receive a limited number of NK cells, extensive proliferation becomes crucial for efficient antiviral function. Overall, the results from the adoptive transfer experiments provide important insight into the role of TNFR2-mediated proliferation in enabling NK cells to effectively limit viral replication.

In summary, we identified that the TNFα-TNFR2 pathway acts as a metabolic switch that skews the metabolic balance to aerobic glycolysis in NK cells. Since NK cells are the first proliferating lymphocyte population during infection and inflammation and proliferation is hardwired to effector function, the effect of TNFα on NK cells is critical to our understanding of NK cell antiviral and antitumor activity. In addition, NK cells are being exploited for adoptive immunotherapy, including chimeric antigen receptor (CAR)-NK cells [56, 57]. The current use of enormously expanded NK cells for NK cell-mediated cancer immunotherapy highlights the potential impact of TNFR2 signaling in ensuring the extensive proliferation and expansion of NK cells, which could be crucial for the development of effective immunotherapeutic strategies. Thus, our results highlighting the TNFα-TNFR2 axis in NK cell proliferation and metabolism expand our understanding of the pleiotropic effect of TNFα on NK cells.

## Limitations and future directions

Our adoptive studies using immunocompromised mice were conducted to investigate NK cell functions without the influence of other cells. It is important to acknowledge that membrane-bound TNFα presented to NK cells by other immune cells plays an important role. This aspect was not included in our study due to the absence of intact immune cell interactions in the immunocompromised mouse models. Furthermore, it is worth noting that there might be unexpected interactions between the adoptive cells and the host cells, which could not be fully investigated using the employed mouse models. Considering these limitations, further research utilizing appropriate models with intact immune cell interactions and exploring the influence of membrane-bound TNFα on NK cell function would provide a more comprehensive understanding of the mechanisms involved in regulating NK cell responses during virus infection. The observation of modestly increased activation of pathways linked to NK cell-mediated immunity in TNFR2-deficient mice, despite decreased proliferation and function, presents an unexpected finding. One possible explanation for this phenomenon is that TNFR2 signaling may serve as a negative regulator of the expression of NK cell receptors and effector molecules. The underlying mechanism behind this upregulation is currently unclear and warrants further investigation.

Our study raises several important questions that remain to be answered. First, we need to investigate the compensatory mechanisms involved in regulating TNFR2 expression on NK cells in *MyD88*^−/−^ and *TRIF*^−/−^ mice during MCMV infection. Second, it is crucial to determine whether TNFR2 signaling plays a role in NK cell exhaustion during chronic conditions. Understanding the involvement of TNFR2 in regulating NK cell exhaustion could provide insights into potential therapeutic strategies to mitigate exhaustion and enhance NK cell responses in chronic infections or diseases. Third, exploring the possibility of upregulating TNFR2 expression in NK cells via CAR technology is an intriguing avenue for adoptive immunotherapy. Investigating whether overexpression of TNFR2 can increase the activity of NK cells for more effective immunotherapy approaches could have important clinical implications. Finally, it is essential to determine the translatability of our study to human immunology. While our findings provide valuable insights into the role of TNFR2 in regulating mouse NK cell responses, further research is needed to investigate the relevance and applicability of these findings in human NK cells and their interactions with TNFR2 signaling. Addressing these open questions will contribute to a deeper understanding of the mechanisms underlying TNFR2 regulation in NK cells, its implications in various disease contexts, and potential therapeutic strategies.

## Methods

### Mice

Wild-type C57BL/6 mice were purchased from Charles River. The NKp46iCre knock-in mouse was a generous gift from Dr. Eric Vivier (Centre d’Immunologie de Marseille-Luminy, Marseille, France) [58]. *Il18r1* floxed mice were a kind gift from Dr. Giorgio Trinchieri (Cancer and Inflammation Program, Center for Cancer Research, National Cancer Institute, NIH, Frederick, MD, USA) [24]. The CD45.1 Ly49H-deficient mice were a gift from Dr. Silvia Vidal (McGill University) [59]. Mice deficient in *TNFR1* (#003242), *TNFR2* (#002620), *MyD88* (#009088), *TRIF* (#005037), NSG (#005557), or *Rag2*^−/−^*Il2rg*^−/−^ (#014593) were purchased from Jackson Laboratory. Mice deficient in *MyD88* and *TRIF* were generated by breeding *MyD88*^−/−^ and *TRIF*^−/−^ mice. All mice were bred and kept in the specific-pathogen-free animal facility at the University of Ottawa in agreement with the guidelines and regulations of the Canadian Council on Animal Care. All procedures were approved by and conducted in accordance with the animal guidelines of the University of Ottawa. All mice used for experiments were between the ages of 6–12 weeks old.

### LPS injection and MCMV infection

C57BL/6 mice were intraperitoneally injected with 50 µg of LPS in PBS. MCMV stocks of the Smith strain were generated in our laboratory from the salivary glands of infected BALB/c mice. Mice were intraperitoneally challenged with MCMV. To determine TNFR expression on NK cells, C57BL/6, *MyD88*^−/−^, and *MyD88*^−/−^*TRIF*^−/−^ mice were intraperitoneally challenged with 3000 PFU MCMV. To determine NK cell proportions in immunocompetent B6 and *TNFR2*^−/−^ mice on Day 4 of MCMV infection, mice were intraperitoneally infected with 3000 PFU MCMV. For the adoptive transfer experiment in vivo, CD45.1 Ly49H-deficient mice were intraperitoneally infected with 3000 PFU MCMV. For the adoptive transfer experiment in vivo, *Rag2*^−/−^*Il2rg*^−/−^ mice were intraperitoneally infected with 5000 PFU MCMV.

### MCMV titer determination

To measure the viral titers, livers from infected mice were homogenized by MagNALyser (Roche Applied Science), and the lysates were diluted and overlaid on mouse embryonic fibroblast cells for 1 h at 37 °C in 2% DMEM (DMEM supplemented with 2% FBS, 1× penicillin/streptomycin, 2 mM L-glutamine, 10 mmol HEPES, and 50 μmol 2-mercaptoethanol). After 1 h of incubation, the virus was removed from the monolayers by aspiration. The monolayers were overlaid with 1 part of DMEM containing 2% low melting agar mixed with 3 parts of 13.5% DMEM (DMEM supplemented with 13.5% FBS, 1× penicillin/streptomycin, 2 mM L-glutamine, 10 mmol HEPES, and 50 μmol 2-mercaptoethanol). Three days later, the cells were fixed with 10% formalin for 10 min and stained with 1% crystal violet for 10 min. Plaques were counted and represented as log PFU/g of organs.

### Lymphocyte isolation, NK cell purification and in vitro culture

Spleens were harvested, and a single-cell suspension was generated following red blood cell lysis and filtration through a 70-μm filter. NK cells were enriched from the spleen by negative selection using the MojoSort Mouse NK cell isolation Kit (BioLegend). The purity of enriched NK cells was >60%. In certain experiments, enriched NK cells were sorted with a Sony SH800 cell sorter, and the purities of sorted NK cells were >95%. NK cells were cultured in RP-10 media (RPMI-1640 medium containing 10% FBS, 1X penicillin/streptomycin, 2 mM L-glutamine, 10 mmol HEPES, 50 μmol 2-mercaptoethanol) for the indicated times in the presence of recombinant human IL-2 (obtained from NCI Preclinical Repository). Purified NK cells were cultured for 24–48 h with different cytokines at the indicated concentrations: IL-2 (100 U/ml), IL-18 (20 ng/ml), and TNFα (30 ng/ml). BAF cells expressing m157-glycoprotein were provided by Dr. Wayne Yokoyama (Washington University in St. Louis).

### Antibodies and flow cytometry

The following mAbs were used: anti-CD3 (17A2 and 145-2C11), anti-TCRβ (H57-597), anti-CD49b (DX5), anti-CD43 (1B11), anti-Ly49H (3D10), anti-CD25 (PC61), CellTrace Violet stain, anti-phospho-S6 (S235/S236) (CUPK43K), anti-CD45.1 (A20), and anti-CD45.2 (104) from eBioscience; anti-NK1.1 (PK136), anti-Ki-67 (B56), anti-CD69 (H1.2F3), anti-CD120a (55R-286), anti-CD120b (TR75-89), anti-IFN-γ (HXG1.2), anti-CD107a (1D4B), anti-NFκB-p65 (pS529), and anti-BrdU (3D4) from BD Biosciences; anti-CD71 (RT7217), anti-CD98 (RL388) from BioLegend; and Live/Dead Fixable Yellow Dead Functional grade TNFα (MP6-XT22) and NKp46 (29A1.4) monoclonal antibodies were obtained from eBioscience. Intracellular staining of Ki-67 was carried out using a Foxp3 staining kit (eBioscience). Cells were acquired using BD LSRFortessa or ThermoFisher Attune NxT and analyzed using Kaluza 1.3 Analysis software (Beckman Coulter) or FlowJo (V10). Control anti-mouse IgG2a kappa isotype antibodies or unstained cells for a particular antibody were used as FMO. Intracellular staining with anti-phospho-S6 (S235/S236), anti-CD107a, and anti-IFN-γ antibodies was performed using BD Cytofix/Cytoperm protocols (BD Biosciences).

### OCR and ECAR measurements

Flow-sorted NK cells were cultured in the presence of 100 U/ml recombinant human IL-2 and treated with 30 ng/ml TNFα for 48 h. XF 96-well microplates (Seahorse Bioscience) were precoated with Cell-Tak (Corning) for 2 h before seeding the NK cells on the plate for real-time analysis of the ECAR and OCR. A total of 150,000 NK cells were cultured per well, and various inhibitors were added (Agilent Seahorse XF Cell Mito Stress Test Kit and Seahorse XF Cell glycolysis Stress Test) at the following concentrations: oligomycin (2 µM), carbonyl cyanide p-(tri-fluromethoxy)phenyl-hydrazone (5 µM), rotenone (100 nM), antimycin (4 µM), 2-deoxyglucose (30 mM) and glucose (5 mM), which allowed the accurate calculation of oxidative phosphorylation (OCR) and glycolysis (ECAR).

### Glucose uptake assay

A total of 5 × 10^5^–10^6^ spleen cells/ml were washed with PBS and incubated for 15 min in RPMI-1640 without glucose (Corning) supplemented with 10% dialyzed serum (Thermo Fisher Scientific), 2 mM L-glutamine, 1 mM HEPES, 1% penicillin/streptomycin and 50 µmol 2-mercaptoethanol at 37 °C. Cells were incubated for 1 h in glucose-free medium with 50 µM 2-NBDG (Life Technologies) at 37 °C. Cells were washed twice with PBS and stained for NK1.1, DX5, TCRβ, and Fixable Yellow Live/Dead (Invitrogen) on ice for 25 min before being analyzed using flow cytometry.

### RNA sequencing

Ly49H^+^ NK cells from CD45.1-wild-type and CD45.2-TNFR2 KO mice were cotransferred into NSG mice. One day later, mice were infected with MCMV. On Day 3.5 postinfection, spleens were harvested, and a single-cell suspension was generated followed by sorting of Ly49H^+^ (CD45.1 or CD45.2) cells with a Sony SH800 instrument. RNA was extracted using the PureLink RNA mini kit (Thermo Fisher).

A poly-A enriched (stranded) library was generated and subjected to 100 bp paired-end sequencing using NovaSeq6000 performed at Genome Quebec in Montreal, Canada. Fastq files were trimmed using Trimmotmatic (v.1.4) and aligned to the mouse assembly (mm10) using Kallisto (v.0.46.1). Gene level quantification was performed using Tximport (v.1.26.1). Differential expression was computed using DESeq2 (v.1.38.3). Gene ontology overrepresentation analysis was performed using clusterProfiler (v.4.6.2) on differentially expressed genes with an adjusted *p* value < 0.05 and a cutoff of log2-fold change >0.5 or <−0.5 for upregulated or downregulated genes, respectively. Figures were generated using ComplexHeatmap (v.2.14.0) and ggplot2 (v.3.4.0). The RNA-seq data have been deposited at GEO with accession number GSE235545.

### Histological analysis

To evaluate the extent of inflammation at the histological level, the major lobe of each mouse liver was cut and fixed in neutral buffered 10% formalin (for 72 h) and embedded in paraffin blocks. Tissue sections of 4 µm thickness were stained with hematoxylin and eosin (H&E staining). Stained slides were scanned using a Zeiss Axio Scan Z1 (objective ×20) to obtain a complete view of the entire tissue section. The images were thoroughly evaluated using ZEN lite (version 3.3) software to select a representative liver area of 8 mm^2^ for counting the number of inflammatory foci. Each inflammatory focus was defined as a cluster of 6–60 nucleated cells following an already established method [60, 61]. The numbers of inflammatory foci in both groups were counted, and the results are presented as the mean ± SEM. All tissue processing, staining, and imaging were performed by the Louise Pelletier Histology Core Facility (Department of Pathology and Laboratory Medicine, University of Ottawa, RRID: SCR - 021737).

### Statistical analysis

The mean values in the experiment were tested by ANOVA. If the ANOVA rejected the null hypothesis of the same means among the conditions (*p* < 0.01), multiple comparisons were performed between selected pairs of means by a two-tailed unpaired t test (*p < 0.05, **p < 0.01, ***p < 0.001) using Prism Version 8 (GraphPad Software).

### Supplementary information

Supplementary Figures

## Data Availability

The RNA-seq data have been deposited at GEO with accession number GSE235545.
