# Supplementary material for: The TNFα/TNFR2 axis mediates natural killer cell proliferation by promoting aerobic glycolysis
Source: Cell Mol Immunol. 2023 Aug 9;20(10):1140–55. doi: 10.1038/s41423-023-01071-4 (PMC10541863; doi:10.1038/s41423-023-01071-4)
Supplement: Supplementary file 1 — Supplementary Figures [file 41423_2023_1071_MOESM1_ESM.pdf]

The TNFα/TNFR2 Axis Mediates Natural Killer Cell Proliferation By Promoting Aerobic Glycolysis

Supplementary Figure 1:

Gating strategy to identify NK cells

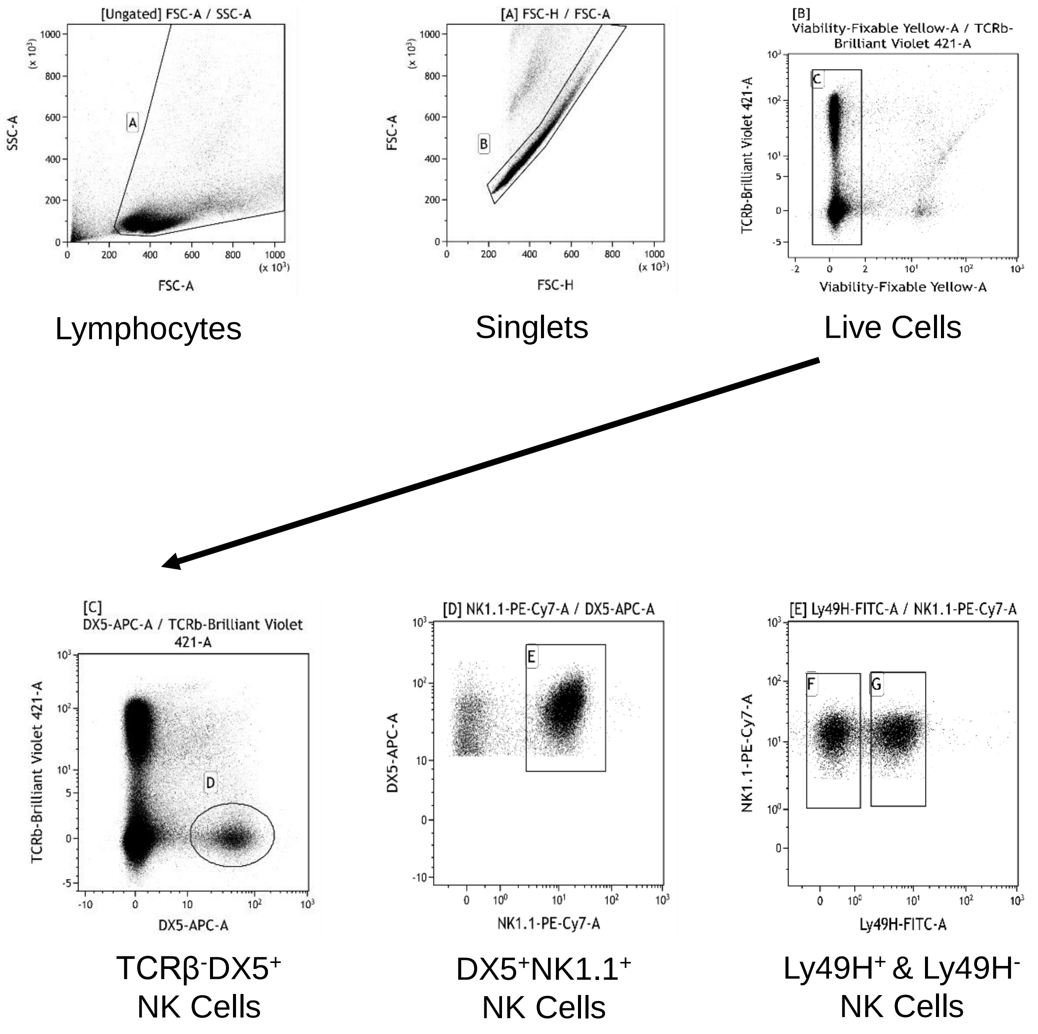

Gating strategy to identifying NK cells

Supplementary Figure 2:

Signaling through IL-18 is dispensable on TNFR2 upregulation during MCMV infection

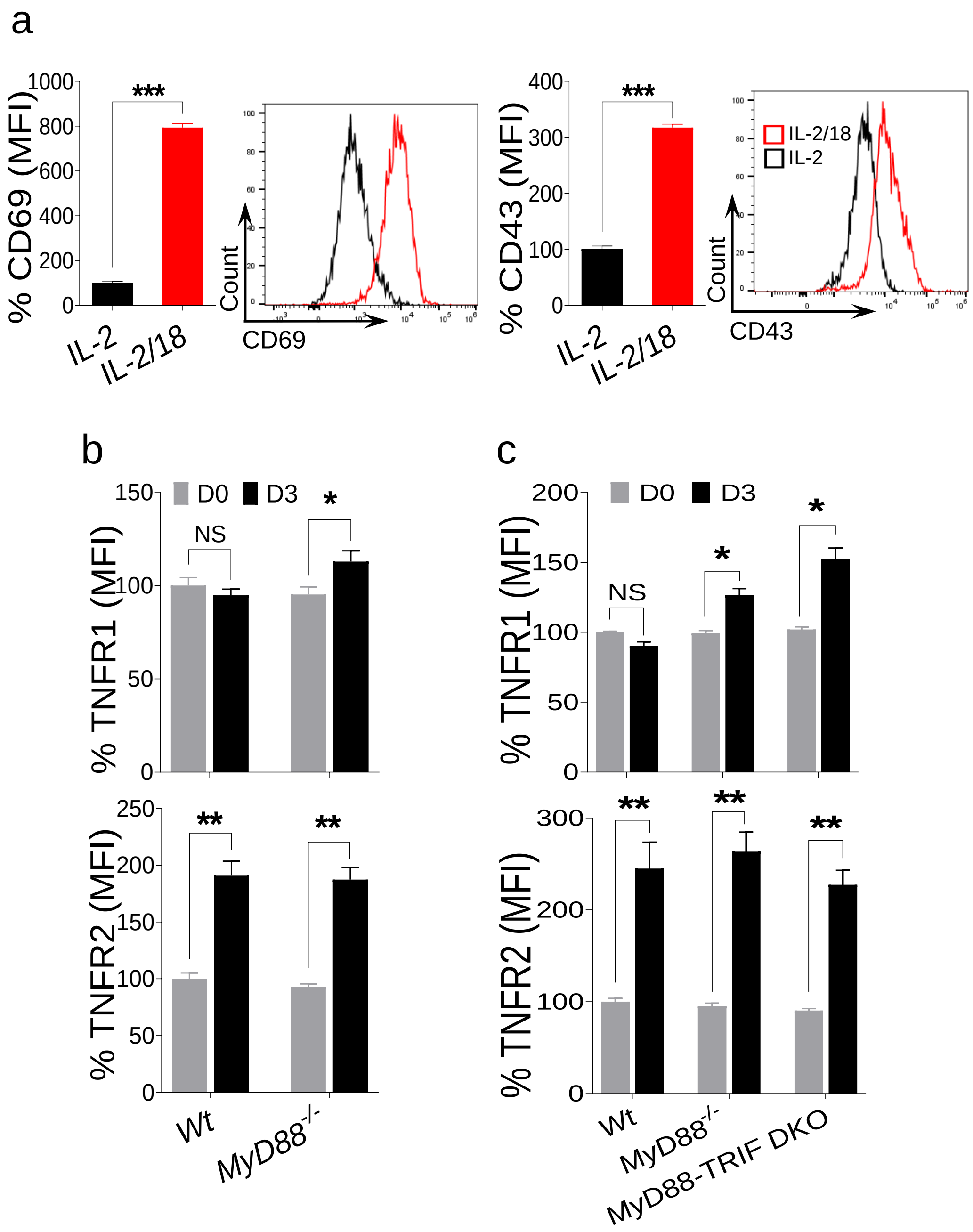

(a) NK cells were enriched from the spleens of naive B6 mice and stimulated with TNFα *ex vivo.* 100U/ml of rhIL-2 was added to maintain NK cell survival. The representative graphs depicts the MFI of CD69 and CD43 expression. **(b)** B6 and *MyD88*$^{-/-}$, **(c)** B6, *MyD88*$^{-/-}$ and *MyD88-TRIF* double knockout is presented. Mice were either left untreated or infected with 3,000 PFU MCMV intraperitoneally and analyzed on the indicated day for the expression of TNFR1 and TNFR2 on splenic NK cells. The MFI expression is presented in percentage relative to the MFI of NK cells from controls as 100. Data are from one experiment representative of two independent experiments, with at least four mice per group. Data represent mean ± SD. $*p < 0.05$; $**p < 0.01$; $***p < 0.001$; *ns*, non-significant.

Supplementary Figure 3:

TNFα promotes activation and proliferation of NK cells

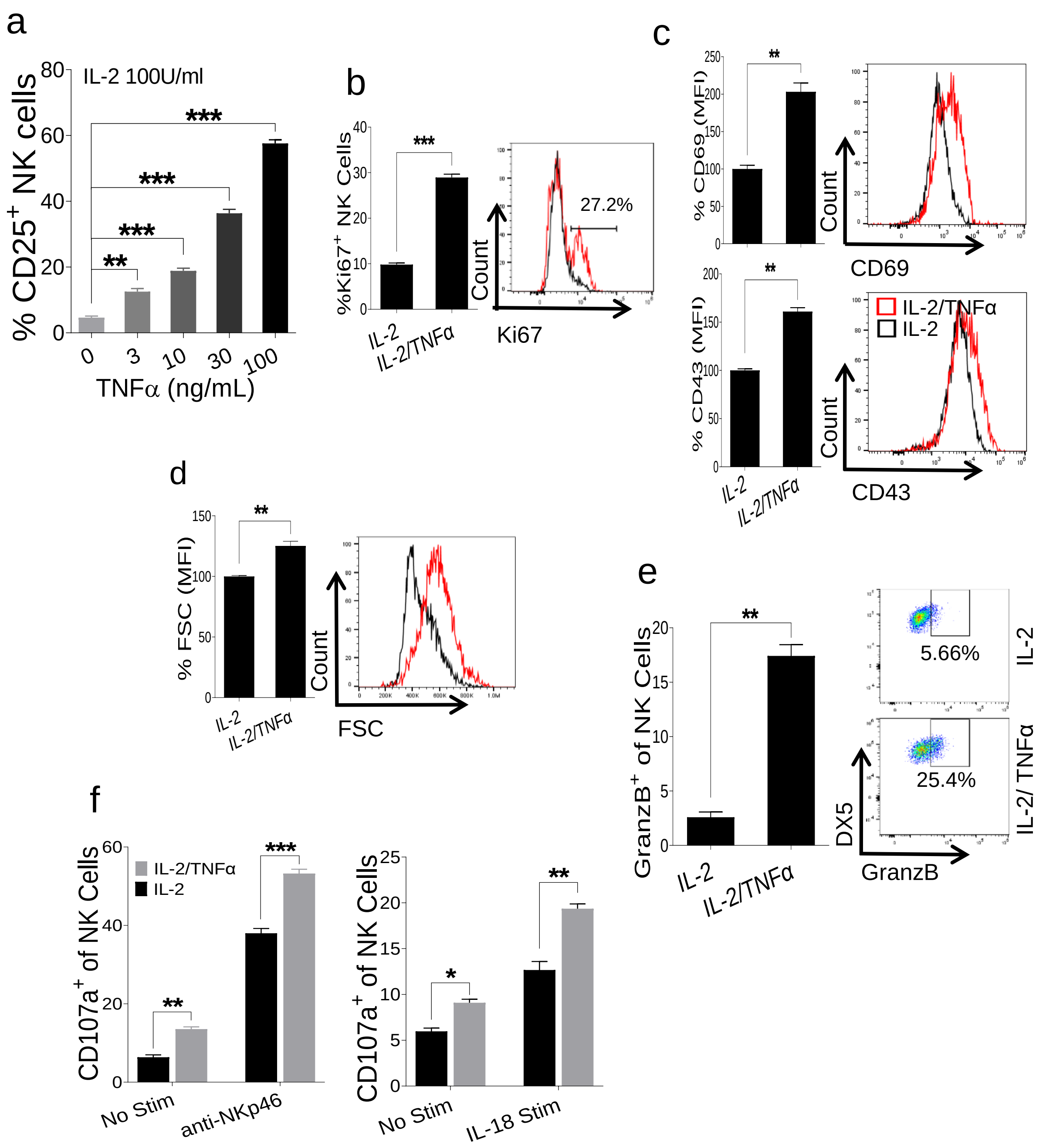

NK cells were enriched from the spleens of naive B6 mice and stimulated with TNFα *ex vivo* for **(a)** 3 days or **(b-e)** 48 hours. 100U/ml of rhIL-2 was added to maintain NK cell survival. The percentage of **(a)** $CD25^+$ cells **(b)** Ki67 $^+$ among the total NK cells population. The MFI of **(c)** CD69 and CD43, **(d)** cell size and **(e)** proportion of granzyme B^+ cells among the total NK cell population were determined. **(f)** Enriched NK cells from naive B6 mice were stimulated with TNFα *ex vivo* for 18 hours followed by stimulation with plate coated anti-NKp46 or IL-18 for additional 5 hours and the proportion of $CD107a^+$ cells among the total NK cell population was measured. The MFI expression is presented in percentage relative to the MFI of IL-2 stimulated NK cells as 100. Data are from one experiment representative of three independent experiments, with at least two replicates per group. Data represent mean ± SD. **p* < 0.05; ***p* < 0.01; ****p* < 0.001.

Supplementary Figure 4:

Autocrine TNFα signaling is important for NK cell functions

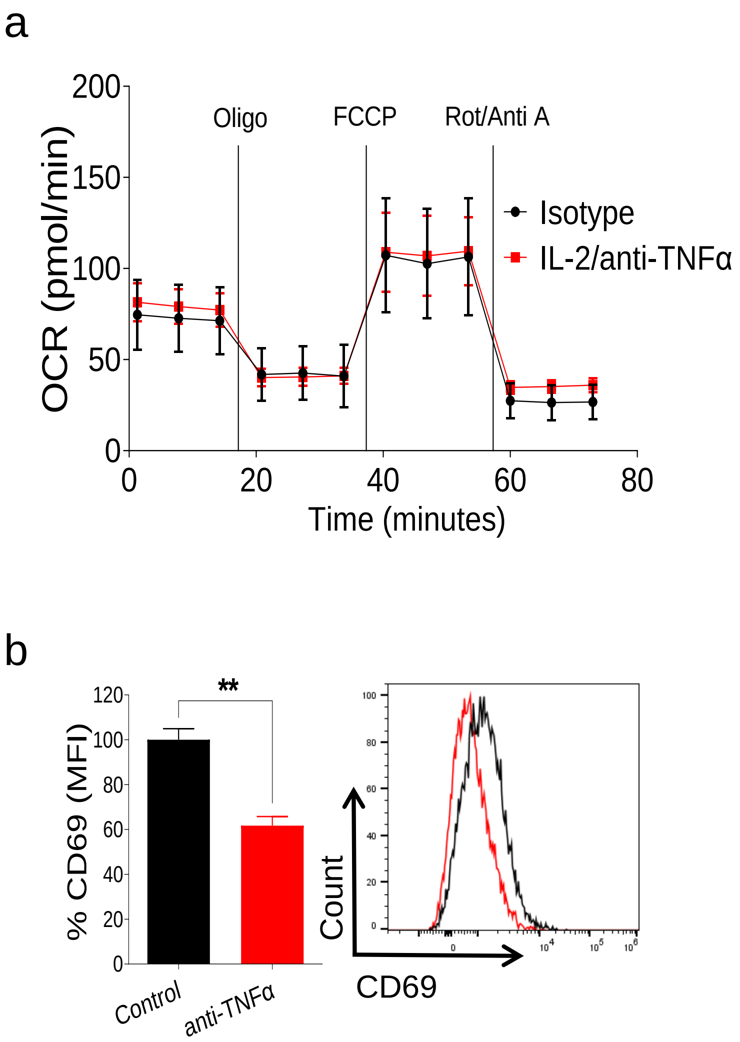

(a) Freshly sorted NK cells from the spleens of naive B6 mice were stimulated with isotype control or anti-TNFα antibodies *ex vivo* for 40 hours and oxygen consumption rate (OCR) was determined. (**b)** Representative graph depicts the expression of CD69 on enriched NK cells treated as in a. 100U/ml of rhIL-2 was added to maintain NK cell survival. Data are from one experiment representative of two independent experiments with at least three replicates per group. Data represent mean ± SD. ** $p < 0.01$.

Supplementary Figure 5:

TNFα signaling is dispensable during NK cells maturation

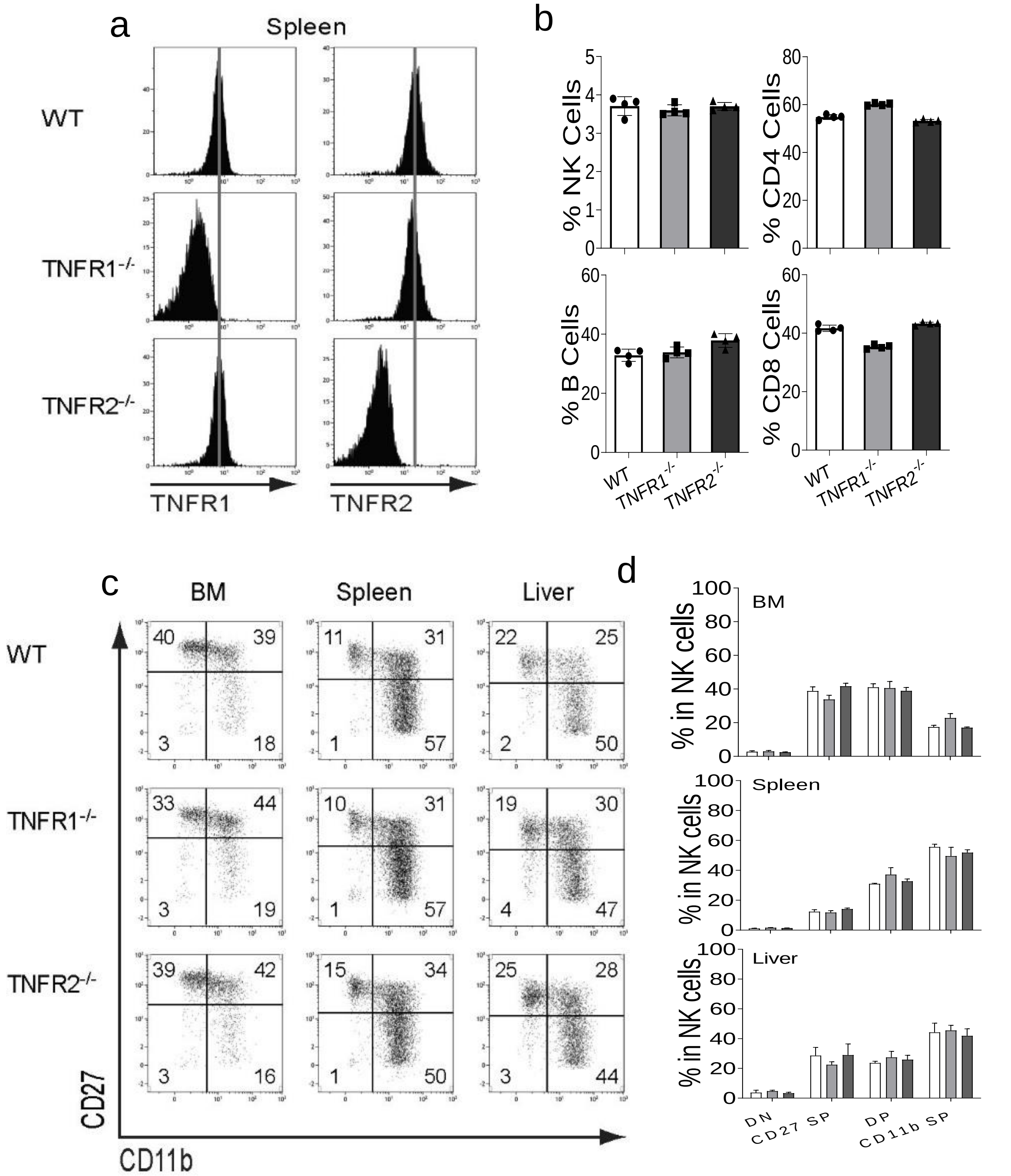

(a) Surface expression of TNFR1 and TNFR2 on NK cells from indicated naive mice. **(b)** Proportions of NK cells, B cells, and T cells in the spleens of naive indicated mice. **(c)** Representative plots, and **(d)** graphs represent the proportion of CD27^{-}CD11b^{-} (DN), CD27^{+}CD11b^{-} (CD27 SP), CD27^{+}CD11b^{+} (DP) and CD27^{-}CD11b^{+} (CD11b SP) populations among NK cells from different organs of indicated mice.

Supplementary Figure 6:

TNFα/TNFR2 axis is critical for the activation, metabolism and effector function in NK cells

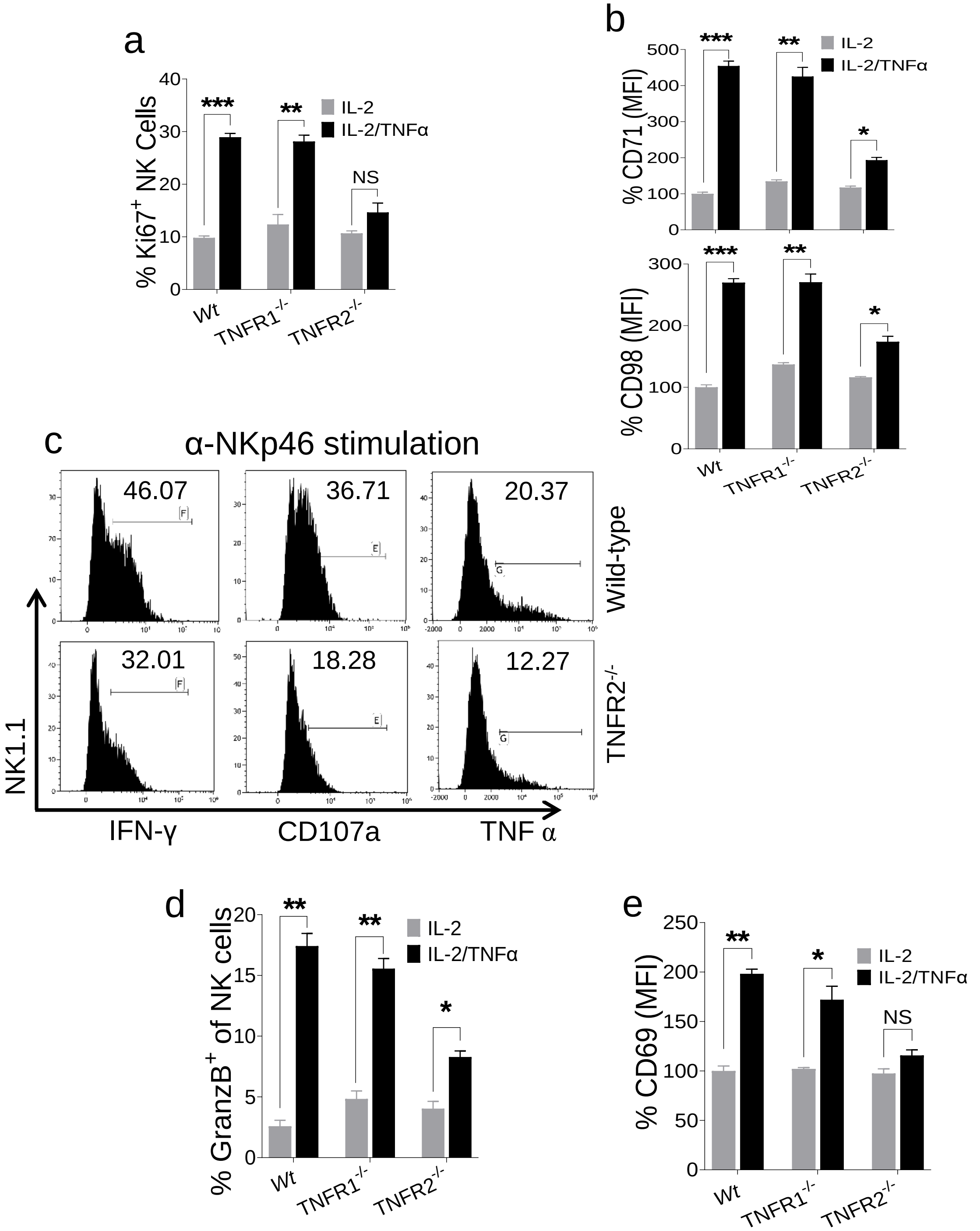

Freshly enriched NK cells from the spleens of naive indicated mice were stimulated with TNFα *ex vivo* for 48 hours in the presence of 100U/ml of rhIL-2. Representative graphs **(a)** represent Ki67$^+$ cells among total NK cell population and **(b)** expression of CD71 and CD98. **(c)** Enriched NK cells from indicated mice were stimulated with plate coated anti-NKp46 for 5 hours and IFN-γ$^+$, CD107a$^+$ and TNFα$^+$ cells were measured among total NK cells population. Cells treated as in **a,** graph represents **(d)** granzyme B$^+$ cells among the total NK cell population, and **(e)** MFI of CD69. The MFI expression is presented in percentage relative to the MFI of IL-2 stimulated NK cells as 100. Data are from one experiment representative of three independent experiments with at least two replicates per group. Data represent mean ± SD. *$p < 0.05$; **$p < 0.01$; ***$p < 0.001$ *ns*, non-significant.

<u>**Supplementary Figure 7:**</u>

TNFα/TNFR2 signaling is vital for NK cell metabolic and functional activity

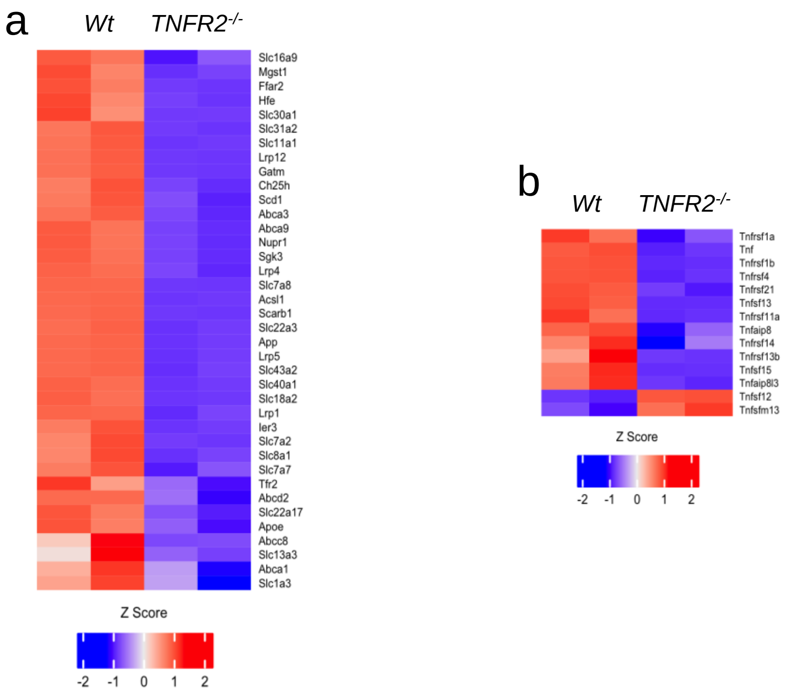

Purified NK cells from wild-type (CD45.1) and TNFR2 (CD45.2) were co-transferred in NSG mice. One day after, mice were infected with MCMV intraperitoneally. Mice were sacrificed on D3.5 following infection and splenic Ly49H$^+$CD45.1$^+$ and Ly49H$^+$CD45.2$^+$ NK cells were flow sorted and used for bulk RNA-sequencing analysis. Heatmap representation of statistically differentially regulated genes **(a)** involved in cellular metabolism, and **(b)** TNFα signaling in NK cells from wild-type versus TNFR2 KO mice. Data are from one experiment performed in duplicate and samples were isolated from the pooled spleen sample generated from 3 infected mice.
